# Electrophysiological signatures decode herbivore-specific defense dynamics and elicitor-induced immune activation in rice

**DOI:** 10.1007/s44297-025-00058-z

**Published:** 2025-09-30

**Authors:** Xinyang Tan, Han Wang, Jing Li, Shuang Zhang, Surui Zheng, Zhichang Zhao, Bo Zhang, Yanyou Wu, Chunqing Zhao, Shuai Li, Rui Ji

**Affiliations:** 1 State Key Laboratory of Agricultural and Forestry Biosecurity, College of Plant Protection, Nanjing Agricultural University, Nanjing, Jiangsu Province, China; 2 Institute of Plant Protection, Jiangsu Academy of Agricultural Sciences, Nanjing, Jiangsu Province, China; 3 School of the Environment and Safety Engineering, Jiangsu University, Zhenjiang, Jiangsu Province, China; 4 State Key Laboratory of Environmental Geochemistry, Institute of Geochemistry, Chinese Academy of Sciences, Guiyang, Guizhou Province, China

**Keywords:** Electrophysiology, Rice, Brown planthopper, Striped stem borer, Elicitor, Interaction

## Abstract

Herbivore infestation triggers complex defense mechanisms in rice, primarily regulated by phytohormones. However, the traditional approach of analyzing plant hormone profiles as an indicator of plant responses to herbivore stress suffers from time-consuming detection, high costs, and destructive sampling of plant tissues. In this study, we propose and validate a novel approach using plant electrophysiological parameters—capacitance, resistance, impedance, and reactance—as a rapid, non-invasive biomarker of rice responses to two major herbivorous pests: the brown planthopper (*Nilaparvata lugens*, BPH) and the striped stem borer (*Chilo suppressalis*, SSB). Systematic modelling revealed herbivore-specific temporal electrophysiological response dynamics, reflecting perturbations in water and nutrient transport, dielectric substance movement, and metabolic energy flux in infested rice plants. Based on these dynamics, we developed predictive models for rice yield potential, drought resistance, and metabolic adaptability under biotic stress. In parallel, we analyzed phytohormone levels and found that changes in electrophysiological traits were strongly associated with jasmonic acid and jasmonic acid-isoleucine accumulation, indicating that these electrical signals capture key aspects of the plant’s immune status. Beyond detecting herbivore-induced defenses, electrical patterns were also mirrored in transgenic rice overexpressing planthopper-derived elicitors (e.g. Myosin/PDI1), indicating electrophysiological signatures act as defense activation markers, thus enabling high-throughput screening of herbivore elicitors. Together, our results highlight the potential of plant bioelectrical signals as fast, integrative, and scalable indicators of stress responses, with applications in crop protection and elicitor-assisted resistance breeding.

**Supplementary Information:**

The online version contains supplementary material available at 10.1007/s44297-025-00058-z.

## Introduction

Rice (*Oryza sativa* L*.*), a staple crop critical to China’s food security, requires stable production to safeguard national food supplies. Planthoppers emerge as primary constraints to rice productivity where outbreak events impact beyond thirty million hectares and induce yield losses above two million metric tons matching more than one percent of yearly national rice production [1]. The brown planthopper (*Nilaparvata lugens*, BPH), a monophagous piercing-sucking pest (Hemiptera: Delphacidae), inflicts the most severe damage due to its explosive reproductive capacity, migratory behaviour, and exclusive feeding on rice phloem via needle-like mouthparts [2]. Equally devastating, the striped stem borer (*Chilo suppressalis*, SSB), a chewing pest (Lepidoptera: Crambidae), adapts to diverse agroecosystems across China. SSB larvae bore into stems, causing dead hearts, white heads, and yield losses through direct tissue destruction [3].

Upon herbivore attack, rice perceives herbivore-associated molecular patterns (HAMPs) released during insect feeding or oviposition, as well as damage-associated molecular patterns, through plasma membrane-localized pattern recognition receptors. This recognition triggers a cascade of defense-related responses, including elevated cytosolic calcium ion (Ca^2^⁺) concentration, reactive oxygen species (ROS) bursts, activation of mitogen-activated protein kinase (MAPK) cascades, jasmonic acid (JA) biosynthesis, release of herbivore-induced plant volatiles, and the production of trypsin protease inhibitors (TrypPIs) [4]. Several salivary proteins from BPH have been identified as HAMPs, which are specifically recognized by rice and elicit defense responses. For example, NlMLP secreted by BPH into rice tissues induces increased Ca^2^⁺ levels, activates MEK2 kinase and the JA signaling pathway, and leads to cell death and callose deposition [5]; NlSP1 also induces plant cell death, H₂O₂ accumulation, and callose deposition [6]; NlG14 and NlDNAJB9 trigger cell death, ROS bursts, callose accumulation, and activate the JA signaling pathway [7, 8]. In contrast, fewer elicitors have been identified from SSB. However, oral secretions of SSB can induce expression of rice defense genes and activate calcium signaling, MAPK pathways, ROS bursts, JA biosynthesis, and TrypPIs accumulation in rice plants [9]. Additionally, IRP1 was found in SSB frass, and can stimulate JA and jasmonic acid-isoleucine (JA-Ile) production, MAPK cascades, and trypsin protease inhibitor synthesis [10]. Both BPH and SSB infestations activated the JA signal pathway, which is crucial for rice defense against herbivores. Upon BPH infestation, rice rapidly upregulates JA biosynthesis genes (e.g., *OsLOX*) [11, 12], leading to JA and JA-Ile accumulation and activation of downstream defense regulators like *OsMYC2* [13]. Transgenic rice lines with impaired JA signaling exhibit heightened susceptibility upon both BPH [14–17] and SSB attacks [10, 18–20]. Similarly, SSB larval feeding triggers JA bursts, which directly induce trypsin protease inhibitors and secondary metabolites detrimental to larval growth [19, 21]. The SA signaling pathway has been demonstrated to positively regulate aphid resistance in Arabidopsis and tomato [22, 23], while the involvement of SA in rice-herbivores interactions remains contentious. Early studies reported no significant SA fluctuations in BPH- or SSB-infested rice plants [24, 25], and neither silencing rice *OsNPR1* gene (a SA signaling receptor) nor overexpressing SA-degrading *OsNahG* gene altered the egg viability of BPH on rice leaf sheath [14]. However, recent work paradoxically showed that *OsNahG* transgenic plants exhibit reduced resistance to BPH nymphs [26], suggesting SA may modulate indirect defenses or baseline immunity.

Despite the clear regulatory importance of phytohormones, their measurement is technically demanding, time-intensive, and requires tissue destruction and specialized instrumentation [27]. This creates a critical bottleneck in real-time monitoring and rapid screening of herbivore-induced responses, particularly under high-throughput or field conditions. While techniques like chlorophyll fluorescence analysis (OJIP-test) have proven sensitive to systemic stress responses induced by root damage in detecting photosynthetic alterations linked to putative electrical signaling (variation potentials) [28], real-time, continuous monitoring of dynamic defense processes, particularly those involving early signaling events and hormone fluctuations triggered by specific herbivores, demands methodologies with higher temporal resolution and direct links to underlying defense physiology. To overcome these constraints, attention has turned toward electrophysiological approaches, which measure the bioelectrical activity of plants as a non-invasive, high-resolution proxy for physiological state. Since Sinyukhin and Britikov's 1967 demonstration of plant-wide electrical signaling throughout life cycles [29], these signals have been recognized as coordinators of growth, nutrient transport, and environmental adaptation through integration with other signaling systems [30–32]. Characterized by non-stationary stochasticity across species and environments [33], bioelectrical patterns shift detectably under stress. The phospholipid-protein bilayer forms a leaky capacitor structure: intracellular/extracellular fluids act as conductive plates separated by lipid dielectric layers. Stress-induced membrane alterations, particularly ion permeability changes, directly modify these electrical characteristics [34], enabling quantitative stress evaluation through signal analysis. Recent advances have established mechanistic mathematical models correlating electrophysiological dynamics with plant physiological states. For instance, equivalent circuit models (e.g., distributed RC networks) simulate membrane polarization and ion flux to quantify stress-induced changes in cellular integrity, as shown by Gautam et al. using impedance spectroscopy to detect nanomaterial-induced conductivity alterations in Aloe vera [35]. Similarly, differential equation frameworks integrating ion channel kinetics can predict systemic electrical responses, evidenced by Sukhov et al. modeling H⁺-ATPase-driven action potential thresholds under cold stress [36]. Xing et al. utilized physiological impedance within a Nernst equation framework to accurately estimate intracellular water content and its role in photosynthesis under drought stress, proposing that electrophysiological dynamics can be harnessed to predict critical agronomic traits [37]. These models provide a theoretical foundation for using electrophysiological signatures as proxies for plant health status.

Electrophysiological parameters such as resistance, impedance, capacitance, and reactance capture the dynamic conductivity properties of plant cells and tissues, reflecting real-time changes in ion fluxes, membrane integrity, and dielectric substance transport. Recent studies have shown that these parameters respond sensitively to abiotic stresses such as drought and salinity, and increasingly, to biotic interactions as well. For example, electrophysiological assays have been used to differentiate drought tolerance in *Bletilla striata* and *Morus alba*, correlating distinct electrical signatures with water use efficiency and oxidative stress marker [38, 39]. Yudina et al. showed that hyperpolarization electrical signals, induced by moderate heating or light in wheat, directly modulated photosynthetic efficiency, with the signal amplitude and physiological impact being modulated by drought severity [40]. This highlights the potential of specific electrical signatures to serve as "mirrors" reflecting and influencing critical physiological activities like photosynthesis and stress adaptation. Zimmermann et al. identified species-specific differences in systemic electrical signals (action potentials, variation potentials, and system potentials) triggered by *Spodoptera littoralis* and *Manduca sexta* larval feeding [41]. Simmi et al. demonstrated pathogen-responsive electrophysiological signals in tomato plants under controlled environments, where leaf-inoculated fungal pathogens generated detectable stem signals, enabling early disease detection [42]. Nevertheless, the application of plant electrophysiology in the context of biotic stress remains underexplored, particularly in relation to herbivore-induced metabolic reprogramming. Direct evidence linking specific bioelectrical parameters to core defense hormone dynamics, particularly the JA pathway which orchestrates anti-herbivore responses in rice, remains scarce. Considering the positive influence of electrical signals on JA concentration [43], although Teng et al. demonstrated in cotton that Methyl Jasmonate (MeJA) application triggered both electrophysiological responses and enhanced JA/MeJA accumulation alongside defensive volatile emission [44], a systematic investigation quantifying the temporal correlation between key electrophysiological indices and JA/JA-Ile levels during live herbivore infestation in a major crop like rice is lacking.

In this study, we hypothesize that electrophysiological parameters, particularly impedance- and reactance-related indices, can reflect herbivore-induced changes in rice metabolism with sufficient sensitivity and specificity to serve as proxies for traditional phytohormones profiling. Using a controlled infestation framework with BPH and SSB, we systematically characterized bioelectrical responses across multiple time points. To mechanistically link electrical traits with underlying metabolic and defense processes, we constructed models integrating electrical properties with indicators of water and nutrient transport, energy metabolism, and hormone accumulation. Importantly, by ectopically expressing planthopper salivary elicitors (e.g. Myosin [45] and PDI1 [46]) in transgenic rice, we successfully recapitulated electrical and hormonal response patterns typical of herbivore feeding. Electrophysiological monitoring can capture herbivore-induced defense states and simulate elicitor responses without actual pest attack, enabling bioelectrical traits as surrogates for screening herbivore-derived or synthetic elicitors. Together, our findings establish a theoretical and empirical framework for real-time, hormone-independent electrical phenotyping of rice defense status, opening new avenues for rapid, scalable, and non-destructive assessment of crop resistance traits.

## Materials and methods

### Plant growth and insect rearing

The japonica rice cultivar Xiushui 11 (XS11) was cultivated under controlled environmental conditions (28 ± 1 °C, 70 ± 5% relative humidity, 16 h: 8 h light/dark cycle) using climate-controlled culture chambers (GXM-358B, Ningbo Jiangnan Instrument Factory, China). Insect colonies of *N. lugens* (brown planthopper, BPH) and *C. suppressalis* (striped stem borer, SSB) were originally collected from rice paddies in Jiangsu Academy of Agricultural Sciences (32.03°N, 118.80°E), with subsequent laboratory domestication. BPH populations were maintained on XS11 seedlings in separate chambers under equivalent thermal conditions but elevated humidity (80 ± 5%) [24], while SSB colonies were reared through > 20 generations using artificial diet in sterilized containers [47].

### Herbivores infestation of rice plants

**Fig. 1:**
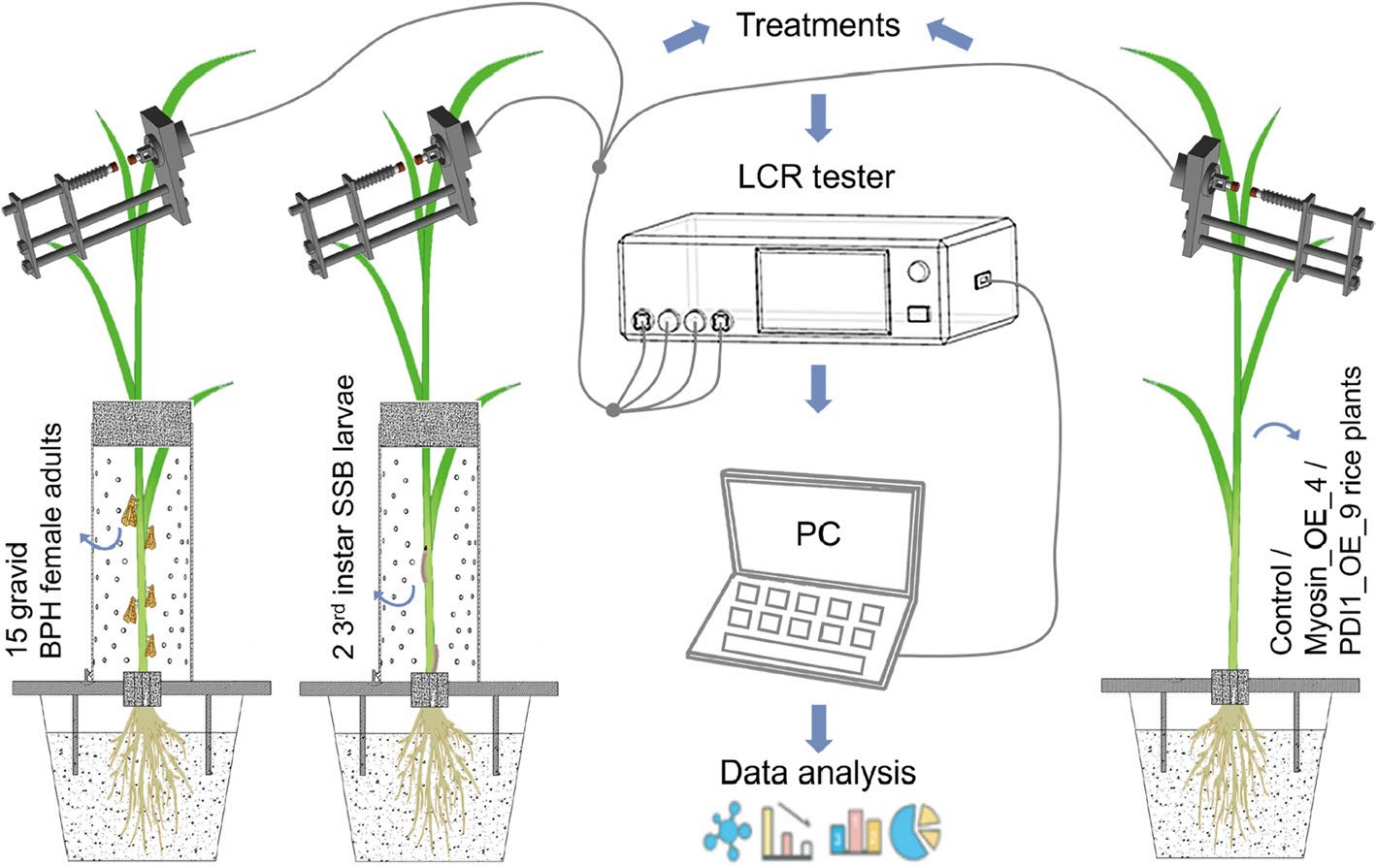
Experimental methods and schematic of the experimental setup. The BPH infestation treatment involved introducing 15 gravid BPH female adults into the experimental setup. The SSB infestation treatment consisted of gently placing two 3^rd^-instar larvae onto rice stems using a fine brush. Rice plants were cultivated individually in pots containing nutrient soil. A perforated glass cylinder was used to confine the insects, with both ends sealed by cotton plugs to prevent BPH and SSB escape. Leaf measurements were conducted using the sensor probe of an LCR tester (Model 3532–50, Hioki, Nagano, Japan). A PC (ThinkPad 1430, Lenovo, Beijing, China) was connected to the LCR tester for data recording and analysis

Forty-five-day-old rice plants (japonica cv. XS11) were systematically subjected to herbivores infestation under controlled laboratory conditions (28 ± 1 °C, 70 ± 5% relative humidity). For BPH treatment group, 15 gravid BPH female adults were confined to the rice leaf sheath using mesh-covered glass cylinders to prevent escape, allowing them to feed continuously for 3 h (BPH_3 h), 8 h (BPH_8 h), and 24 h (BPH_24 h) [24] (Fig. 1). For SSB treatment groups, two 3^rd^-instar SSB larvae were placed on the leaf sheath of each rice plant and allowed to bore into and feed on the plant for 3 h (SSB_3 h) and 8 h (SSB_8 h) [25] (Fig. 1). For both BPH and SSB treatment groups, cotton pads were placed around the plant base to prevent insects from burrowing into the soil. Following designated feeding intervals, plants were immediately transferred to climate-controlled chambers for electrophysiological parameter assessments. The experiment was performed three times with five plant samples per replicate. Simultaneously, samples treated under same intervals were flash-frozen in liquid nitrogen and stored at −80 °C for subsequent phytohormone profiling.

### Establishment of electrophysiological model

The physiological electrical parameters of plant leaves, including capacitance (C), resistance (R), impedance (Z), intrinsic capacitive reactance (Xc), and intrinsic inductive reactance (Xl), were measured using an LCR meter (Model 3532–50, HIOKI, Nagano, Japan) following the methodology established [48, 49]. These parameters exhibit dynamic responses to changes in cellular electrolyte concentration within rice leaf sheaths. During measurement, the leaf sheath sample was positioned between parallel electrode plates to form a parallel-plate capacitive sensor configuration. Variations in clamping force (serving as controlled external stimuli) were found to modulate cell membrane permeability, triggering instantaneous alterations in cellular solute concentrations. This mechano-stimulated ionic redistribution consequently induced measurable changes in all five electrical parameters. The measurements were conducted under standardized conditions with an excitation frequency of 3 kHz and an applied voltage of 1.5 V, ensuring consistent electrical characterization across experimental trials.

C model:

$$\mathrm{C} = {\mathrm{x}}_{0}+ \mathrm{hF}$$

Where ${\mathrm X}_0=\frac{2\Delta\mathrm H}{\mathrm U^2}$, $\mathrm{h} = \frac{\mathrm{2d}}{{\mathrm{U}}^{2}}$, ∆H is the internal energy of the leaf system, U is the voltage parameter of the test setup, and C is the physiological capacitance of rice.

R model:

$$\mathrm{R} = {\mathrm{y}}_{1}\mathrm{+}{\mathrm{k}}_{1} \, {\mathrm{e}}^{-{\mathrm{b}}_{1}{\mathrm{F}}}$$

where ${\mathrm{y}}_{1}\mathrm{=}\frac{{\mathrm{f}}_{0}}{{\mathrm{C}}_{\mathrm{T}}}$, ${\mathrm{k}}_{1}\mathrm{=}\frac{{\mathrm{f}}_{0}}{{\mathrm{C}}_{\mathrm{T}}}{\mathrm{e}}^{\frac{{\mathrm{n}}_{\mathrm{R}}{{\mathrm{F}}}_{0}{{\mathrm{E}}}^{0}}{{\mathrm{R}}_{0}{\mathrm{T}}}}$, ${\mathrm{b}}_{1}\mathrm{=}\frac{\text{d }{\mathrm{n}}_{\mathrm{R}}{{\mathrm{F}}}_{0}}{{\mathrm{a}}{\text{ R}}_{0}{\mathrm{T}}}$, d, a, E^0^, R_0_, T, n_R_, F_0_, C_T_, and f_0_ are fixed values.

Z model:

$$\mathrm{Z} = {\mathrm{y}}_{2}\mathrm{+}{\mathrm{k}}_{2} \, {\mathrm{e}}^{-{\mathrm{b}}_{2}{\mathrm{F}}}$$

where ${\mathrm{y}}_{2}\mathrm{=}\frac{{\mathrm{J}}_{0}}{\mathrm{Q}}$, ${\mathrm{k}}_{2}\mathrm{=}\frac{{\mathrm{J}}_{0}}{\mathrm{Q}}{{\mathrm{e}}}^{\frac{{{\mathrm{n}}_{\mathrm{Z}}{\mathrm{F}}}_{0}{{\mathrm{E}}}^{0}}{{\mathrm{R}}_{0}{\mathrm{T}}}}$, ${\mathrm{b}}_{2}\mathrm{=}\frac{{\mathrm{d}}{\mathrm{n}}_{\mathrm{Z}}{{\mathrm{F}}}_{0}}{\text{ a}{\text{ R}}_{0}{\mathrm{T}}}$, d, a, E^0^, R_0_, T, n_Z_, F_0_, Q, and J_0_ are fixed values.

Xc model:

$$\mathrm{Xc} = {\mathrm{y}}_{3}\mathrm{+}{\mathrm{k}}_{3} \, {\mathrm{e}}^{-{\mathrm{b}}_{3}{\mathrm{F}}}$$

where ${\mathrm{y}}_{3}\mathrm{=}\frac{{\mathrm{L}}_{0}}{\mathrm{X}}$, ${\mathrm{k}}_{3}\mathrm{=}\frac{{\mathrm{L}}_{0}}{\mathrm{X}}{{\mathrm{e}}}^{\frac{{{\mathrm{n}}_{\mathrm{Xc}}{\mathrm{F}}}_{0}{{\mathrm{E}}}^{0}}{{\mathrm{R}}_{0}{\mathrm{T}}}}$, ${\mathrm{b}}_{3}\mathrm{=}\frac{{\mathrm{d}}{\mathrm{n}}_{\mathrm{Xc}}{{\mathrm{F}}}_{0}}{\text{ a}{\text{ R}}_{0}{\mathrm{T}}}$, d, a, E^0^, R_0_, T, n_Xc_, F_0_, X, and L_0_ are fixed values.

Xl model:

$$\mathrm{Xl} = {\mathrm{y}}_{4}\mathrm{+}{\mathrm{k}}_{4} \, {\mathrm{e}}^{-{\mathrm{b}}_{4}{\mathrm{F}}}$$

where ${\mathrm{y}}_{4}\mathrm{=}\frac{{\mathrm{P}}_{0}}{\mathrm{M}}$, ${\mathrm{k}}_{4}\mathrm{=}\frac{{\mathrm{P}}_{0}}{\mathrm{M}}{{\mathrm{e}}}^{\frac{{{\mathrm{n}}_{\mathrm{Xl}}{\mathrm{F}}}_{0}{{\mathrm{E}}}^{0}}{{\mathrm{R}}_{0}{\mathrm{T}}}}$, ${\mathrm{b}}_{4}\mathrm{=}\frac{{\mathrm{d}}{\mathrm{n}}_{\mathrm{Xl}}{{\mathrm{F}}}_{0}}{\text{ a}{\text{ R}}_{0}{\mathrm{T}}}$, d, a, E^0^, R_0_, T, n_Xl_, F_0_, M, and P_0_ are fixed values.

The specific derivation process was described in [48, 49].

### Acquisition of cell metabolic energy

Rice leaf cell metabolic energy ΔG_R_ (10^−12^ J) based on R, ΔG_Z_ (10^−12^ J) based on Z, ΔG_Xc_ based on Xc, ΔG_Xl_ based on Xl. The specific effective thickness d (10^−12^ m) of rice leaves was calculated based on C, R, Z, Xc, Xl. The unit metabolic energy of plant leaf cell ΔG_R-U_ (J/m) based on R, ΔG_Z-U_ (J/m) based on Z, ΔG_Xc-U_ (J/m) based on Xc, ΔG_Xl-U_ (J/m) based on Xl [49].

### Determination of intrinsic electrophysiological parameters, water metabolism, nutrient metabolism, and leaf metabolic activity parameters based on electrophysiological parameters

Intrinsic electrophysiological parameters: Through equation fitting of parameters C, R, Z, Xc, and Xl in rice leaves under varying clamping forces (F), the intrinsic current parameters IC, IR, IZ, IXc, and IXl can be computationally derived at zero clamping force (F = 0) as follows: IR = y_1_ + k_1_, IZ = y_2_ + k_2_, IXc = y_3_ + k_3_, IXl = y_4_ + k_4_, $\mathrm{IC}=\frac1{2\pi\mathrm{fIXc}}$

Water metabolism indicators: Plant leaf cells can be approximated as spherical structures. The capacitance C of leaf cells can be calculated using the formula for concentric spherical capacitors:

$$\mathrm C=\frac{4\pi\varepsilon{\mathrm R}_1{\mathrm R}_2}{{\mathrm R}_2-{\mathrm R}_1}$$

where C is the capacitance of the concentric spherical capacitor, R_2_-R_1_ represents the membrane thickness. Consequently, the relationship between cell volume Vc and C is expressed as:

$$\mathrm{Vc}=\mathrm\alpha\sqrt{\left(\mathrm C\right)^3}$$

For homologous cells within the same plant tissue or organ, α is a cell-type-specific constant. Since cell volume correlates with water-holding capacity, $\sqrt{{\mathrm{C}}^{3}}$ can characterize leaf intracellular water-holding capacity. Thus, IWHC is calculated as:

$$\mathrm{IWHC} = \sqrt{{\left({\mathrm{IC}}\right)}^{3}}$$

The specific effective thickness d of leaves, reflecting cellular growth dynamics, is directly supported by IWHC. The relative intracellular water use efficiency is therefore defined as:

$$\mathrm{IWUE} = \frac{\mathrm{d}}{{\mathrm{IWHC}}}$$

Based on Ohm’s Law and the equivalence of current to the temporal derivative of capacitive voltage, the intracellular water retention time is derived via integral transformation as the product of capacitance and impedance:

$$\mathrm{IWHT}=\mathrm{IC}\times \mathrm{IZ}$$

The intracellular water transfer rate is calculated by:

$$\mathrm{IWTR} = \frac{\mathrm{IWHC}}{{\mathrm{IWHT}}}$$

Nutrient metabolism indicators: Membrane proteins are critically involved in nutrient transport. The specific nutrient flux is defined as:

$$\mathrm{UNF} = \frac{\mathrm{R}}{{\mathrm{I}}{\mathrm{Xc}}}\mathrm{+}\frac{\mathrm{R}}{{\mathrm{I}}{\mathrm{Xl}}}$$

Given the aqueous dissolution of nutrients, the nutrient transfer rate is conceptually equivalent to IWTR and calculated identically:

$$\mathrm{NTR}=\frac{\sqrt{{\left(\mathrm{IC}\right)}^{3}}}{\mathrm{IC}\times \mathrm{IZ}}$$

Nutrient transport capacity is determined by multiplying UNF and NTR:

$$\mathrm{NTC}=\mathrm{UNF}\times \mathrm{NTR}$$

Binding proteins are most closely associated with active nutrient transport. The specific active nutrient flux is defined as:

$$\mathrm{UAF} = \frac{\mathrm{IXc}}{{\mathrm{IXl}}}$$

Nutrient active transport capacity is derived from UAF and NTR:

$$\mathrm{NAC}=\mathrm{UAF}\times \mathrm{NTR}$$

Leaf metabolic activity parameters: Metabolic capacity exhibits intrinsic coupling with bioelectrical currents. Enhanced bioelectrical activity correlates with optimized metabolic performance. Consequently, we define the metabolic flux of plant leaves as:

$$\mathrm{MF}=\frac{1}{\mathrm{IR}\times \mathrm{IZ}\times \mathrm{IXC}\times \mathrm{IXL}}$$

Active nutrient transport is mechanistically linked to metabolic rate. Elevated active transport capacity directly reflects increased metabolic kinetics. Thus, the metabolic rate is defined as:

$$\mathrm{MR}=\mathrm{IWTR}\times \mathrm{NAC}$$

The relative metabolic activity is derived from both MF and MR through the relationship:

$$\mathrm{MA}=\sqrt[6]{\mathrm{MF}\times \mathrm{MR}}$$

### Generation of planthopper salivary elicitor overexpressing rice lines

Building upon our prior characterization of salivary elicitors from the small brown planthopper (SBPH), a functionally validated protein: Myosin (RZF45242.1) [45] and a BPH functionally validated protein: PDI1 (KU365961.1) [46] were selected for further assays. The Myosin and PDI1 was ligated into the vector pCAMBIA1301 (Cambia, USA) under the control of the CaMV 35S promoter. The T-DNA was introduced into the genome of *Nipponbare* rice using *Agrobacterium tumefaciens* mediated transformation. Homozygous T2 plants were selected using hygromycin resistance and GUS staining.

### Quantification of plant hormones

The collected leaf sheath samples were immediately flash-frozen in liquid nitrogen and mechanically homogenized using a grinding mill with 5-mm steel beads at 30 Hz for 1 min. Precisely 50 mg of the resulting frozen powder was weighed and mixed with 10 μL of isotope-labeled internal standards solution (^2^D6-JA, ^2^D6-JA-Ile, ^2^D4-SA, each at 100 ng/mL concentration) followed by the addition of 1 mL of chilled methanol/water/formic acid extraction solvent (15:4:1, v/v/v). The mixture was vigorously vortexed for 10 min to ensure complete analyte dissolution and subsequently centrifuged at 12,000 r/min for 5 min at 4 °C. The supernatant was carefully transferred, concentrated under nitrogen stream at 35 °C, and reconstituted in 100 μL of 80% (v/v) methanol/water solution. After filtration through 0.22 μm nylon membranes, samples were subjected to LC–MS/MS analysis using an ExionLC™ AD ultra-performance liquid chromatography system hyphenated to a QTRAP® 6500 + triple quadrupole mass spectrometer (Sciex, Framingham, MA). Chromatographic separation was achieved on a Waters ACQUITY UPLC HSS T3 C18 analytical column (1.8 μm particle size, 100 mm length × 2.1 mm internal diameter) maintained at 40 °C. The mobile phase consisted of 0.04% (v/v) acetic acid in ultrapure water (eluent A) and 0.04% (v/v) acetic acid in HPLC-grade acetonitrile (eluent B) delivered at 0.35 mL/min flow rate. A multistep gradient elution program was employed: initial 95% A (0–1.0 min), linear ramp to 5% A over 7 min (1.0–8.0 min), isocratic hold at 5% A (8.0–9.0 min), rapid return to 95% A (9.1 min), and column re-equilibration (9.1–12.0 min). Mass spectrometric detection utilized electrospray ionization operated under polarity switching mode with source parameters optimized as follows: turbo spray temperature 550 °C, curtain gas 35 psi, ion spray voltages + 5500 V (positive mode) and −4500 V (negative mode). Compound-specific declustering potentials and collision energies were calibrated for each target analyte in multiple reaction monitoring mode according to Metware Database standards (https://www.metware.cn/). Quantification was performed by integrating chromatographic peak areas against matrix-matched calibration curves spanning 0.1–500 ng/mL [50]. The experiment was performed three times with six plant samples per replicate.

### Quantitative Real-Time PCR

Total RNA was isolated from plant samples employing the SteadyPure Plant RNA Extraction Kit (Accurate Biology, Hunan, China). Subsequently, reverse transcription was performed on 1 µg aliquots of total RNA using HiScript Reverse Transcriptase (Vazyme, Nanjing, China) to synthesize complementary DNA. Quantitative PCR was performed using the Hieff UNICON® Universal Blue qPCR SYBR Green Master Mix (YEASEN, Shanghai, China) according to the protocol [51]. Transcript levels in the treatment group were compared to the control group using the 2^−ΔΔCt^ method, with normalization based on the reference gene *OsEF1-alpha* [52, 53]. The qPCR experiment was repeated three times. Primer sequences were obtained in former studies [45, 46].

### Statistical analyses

Prior to analysis of variance (ANOVA), normality and homogeneity of variance were assessed using Bartlett’s test. One-way ANOVA was subsequently performed, followed by Duncan’s multiple range test. Statistical significance is indicated as follows: asterisks denote significant differences (* *p* < 0.05; ** *p* < 0.01; ns, not significant; Student’s t-test), while different lowercase letters indicate significant differences among treatments (*p* < 0.05). All statistical analyses were conducted using the IBM SPSS system (https://www.ibm.com/spss). Numerical results are presented as means ± standard errors of the mean (SEM).

## Results

### Herbivore infested-rice physiological capacitance (C), resistance (R), impedance (Z), capacitive reactance (Xc), and inductive reactance (Xl) models

**Fig. 2:**
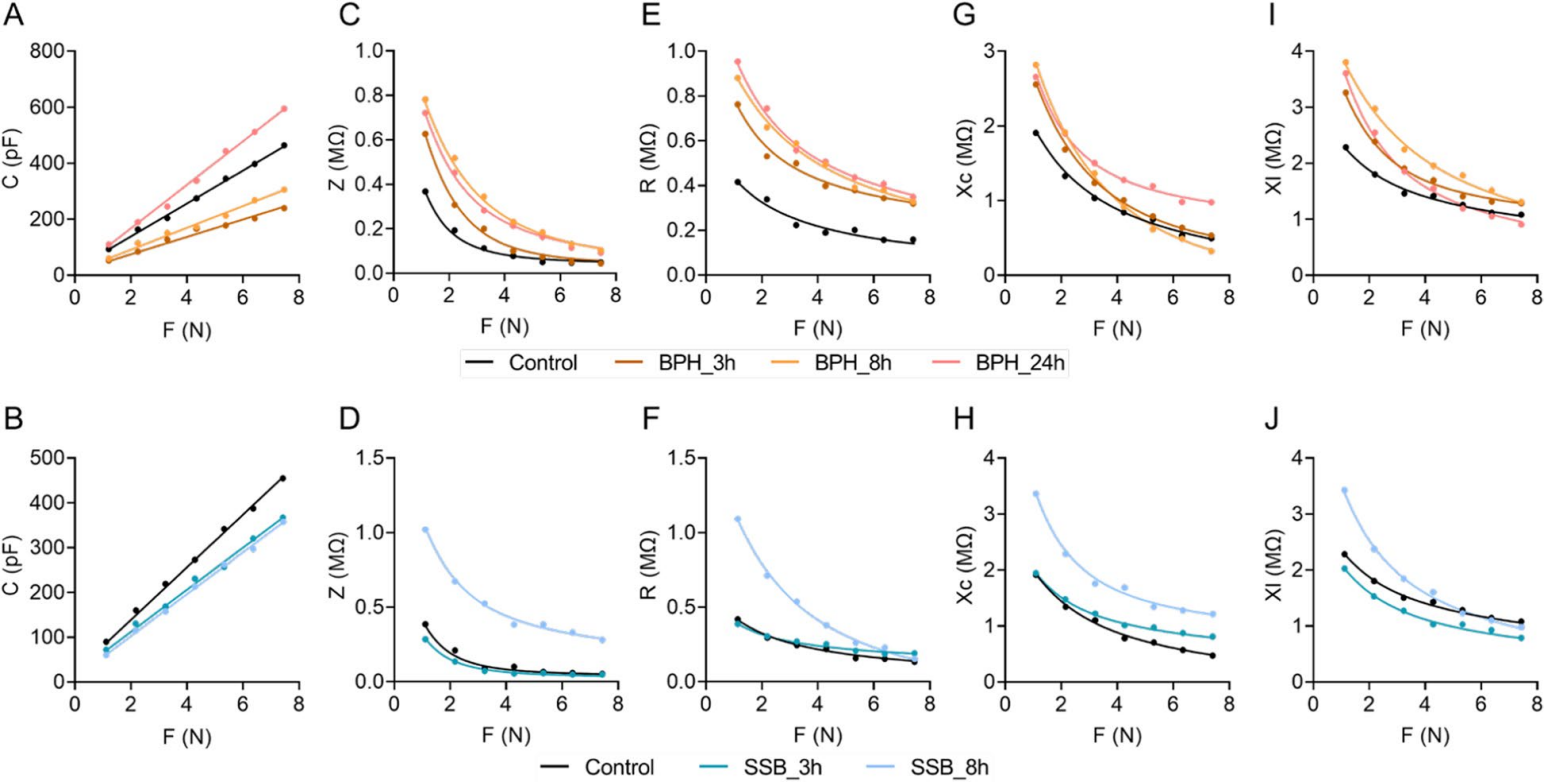
Clamping force-dependent variations in electrical parameters of herbivores-infested rice leaves. Variation of physiological C (**A**, **B**), Z (**C**, **D**), R (**E**, **F**), Xc (**G**, **H**), and Xl (**I**, **J**) of BPH-infested (upper panel) and SSB-infested (bottom panel) rice leaves with clamping force F, respectively. C, physiological capacitance; Z, physiological impedance; R, physiological resistance; Xc, physiological capacitive reactance; Xl, physiological inductive reactance

As the clamping force F increases, the C of the rice leaves increases, and the C changes linearly with the clamping force (Fig. 2A, B). Origin 2018 was used to dynamically fit the experimental data, obtain the parameters of x_0_ and h in the equation C = x_0_ + Hf, obtain the function parameters and equations of the C of the leaves and the clamping force F, and obtain the statistical data of the equation fit. The values of *R*^2^, *n* and *p* values are shown in Table S1. As the clamping force F increases, the R, Z, Xc, and Xl of the rice leaves increases, and the R, Z, Xc, and Xl decreases exponentially with the change of the clamping force F relationship (Fig. 2C-J). Using Origin 2018 to dynamically fit the experimental data, the parameters of y, k and b in the equation $\mathrm{R} = {\mathrm{y}}_{1}\mathrm{+}{\mathrm{k}}_{1} \, {\mathrm{e}}^{-{\mathrm{b}}_{1}{\mathrm{F}}}$, ${\mathrm{Z}}\mathrm{=}{\mathrm{y}}_{2}\mathrm{+}{\mathrm{k}}_{2} \, {\mathrm{e}}^{-{\mathrm{b}}_{2}{\mathrm{F}}}$, ${\mathrm{Xc}}\mathrm{=}{\mathrm{y}}_{3}\mathrm{+}{\mathrm{k}}_{3} \, {\mathrm{e}}^{-{\mathrm{b}}_{3}{\mathrm{F}}}$, and ${\mathrm{Xl}}\mathrm{=}{\mathrm{y}}_{4}\mathrm{+}{\mathrm{k}}_{4} \, {\mathrm{e}}^{-{\mathrm{b}}_{4}{\mathrm{F}}}$ are obtained, and the R, Z, Xc and Xl of the rice leaves are obtained as a function of the clamping force F parameters and equations; at the same time, the equation-fitting statistics R^2^, *n* and *p* values are obtained (Table S2).

### Herbivores infestation induces specific changes in rice electrophysiological parameters

**Fig. 3:**
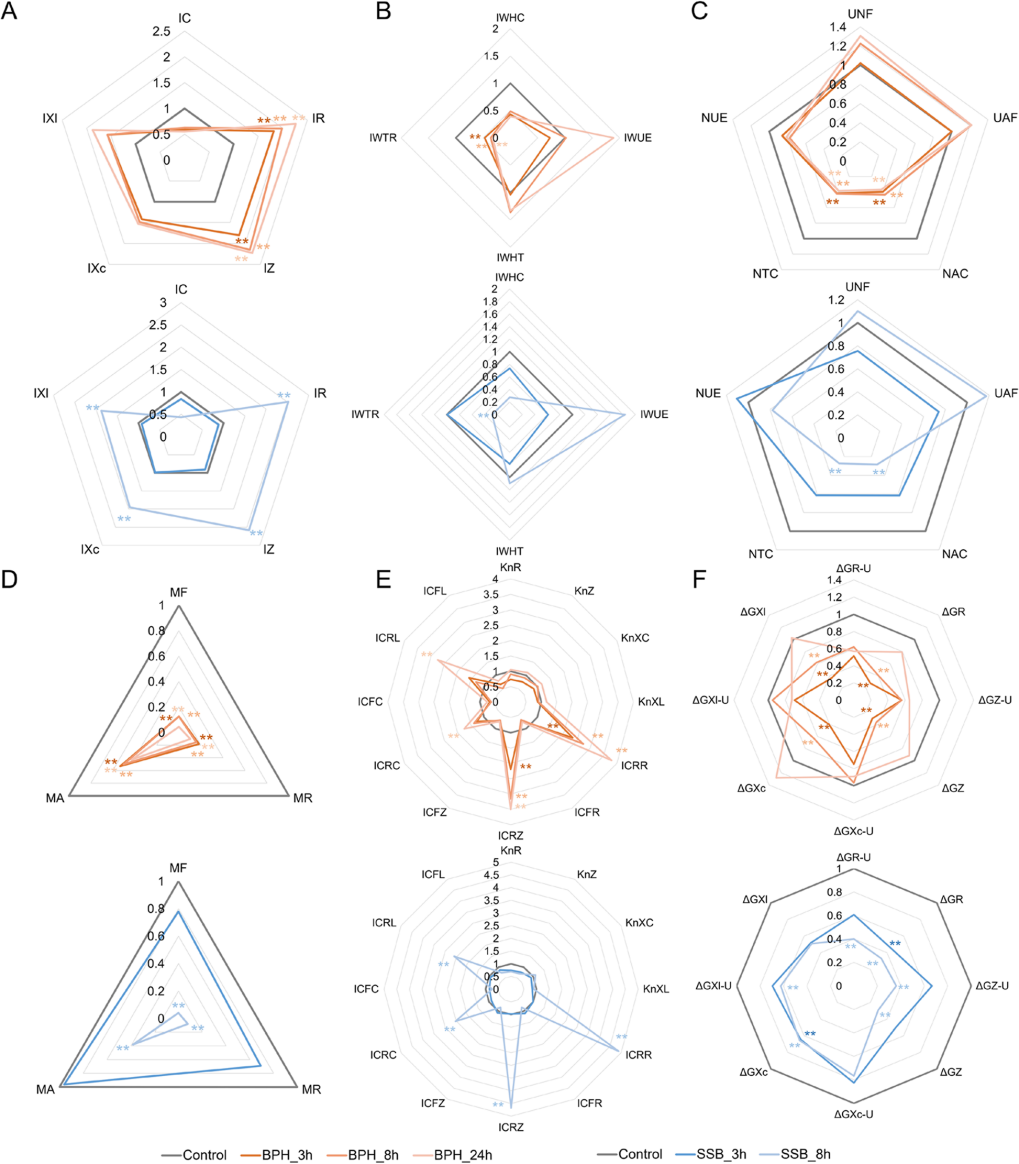
Radar charts of rice physiological parameters under herbivores infestation at different time stages. (**A**-**F**) Electrophysiological parameters (**A**), intracellular water metabolism indicators (**B**), intracellular nutrient metabolism indicators (**C**), leaf metabolic activity indicators (**D**), intracellular dielectric substance transport indicators (**E**), and energy metabolism indicators of various intracellular dielectric substances (**F**) in rice plants subjected to BPH or SSB feeding at different time stages. Upper panels represent the BPH_3h, BPH_8h, and BPH_24h groups. Bottom panels represent the SSB_3h and SSB_8h groups. IC, intrinsic physiological capacitance; IR, intrinsic physiological resistance; IZ, intrinsic physiological impedance; IXc, intrinsic physiological capacitive reactance; IXl, intrinsic physiological inductive reactance; IWHC, intracellular water holding capacity; IWUE, intracellular water use efficiency; IWHT, intracellular water holding time; IWTR, intracellular water transfer rate; UNF, unit nutrient flux; UAF, unit active flux; NAC, nutrient active transport capacity; NTC, nutrient transport capacity; NUE, nutrient use efficiency; MF, metabolic flux; MR, metabolic rate; MA, metabolic activity; Kn_R_, transfer number of dielectric substance in response to physiological resistance; Kn_Z_, transfer number of dielectric substance in response to physiological impedance; Kn_XC_, transfer number of dielectric substance in response to physiological capacitive reactance; Kn_XL_, transfer number of dielectric substance in response to physiological inductive reactance; ICR_R_, intracellular conductive resistance of resistive substance; ICF_R_, intracellular conductive force of resistive substance; ICR_Z_, intracellular conductive resistance of total dielectric substance; ICF_Z_, intracellular conductive force of total dielectric substance; ICR_C_, intracellular conductive resistance of capacitive substance; ICF_C_, intracellular conductive force of capacitive substance; ICR_L_, intracellular conductive resistance of inductive substance; ICF_L_, intracellular conductive force of inductive substance; ΔG_R-U_, unit metabolic energy based on resistance; ΔG_R_, metabolic energy based on resistance; ΔG_Z-U_, unit metabolic energy based on impedance; ΔG_Z_, metabolic energy based on impedance; ΔG_Xc-U_, unit metabolic energy based on capacitive reactance; ΔG_Xc_, metabolic energy based on capacitive reactance; ΔG_Xl-U_, unit metabolic energy based on inductive reactance; ΔG_Xl_, metabolic energy based on inductive reactance. Asterisks indicate significant differences (** *p* < 0.01; * *p* < 0.05; Student’s t-test)

Herbivore-specific temporal dynamics in rice electrophysiological parameters were revealed through comprehensive impedance analysis (Fig. 3, Table S3). Sustained feeding by the phloem-feeding brown planthopper (BPH) progressively enhanced both IR (202.49%) and IZ (206.15%), with significant elevations detected as early as 3 h post-infestation that intensified through 24 h exposure (Fig. 3A). This persistent elevation pattern suggests cumulative bioelectrical interference with resistive cellular components and dielectric substance flow during prolonged piercing-sucking activity. In contrast, striped stem borer (SSB) larvae elicited a delayed but broader-spectrum response, with 8 h infestation simultaneously increasing IR (252.23%), IZ (258.46%), IXc (195.14%), and IXl (198.46%), indicating coordinated disruption of multiple electrical transport mechanisms following mechanical tissue destruction. Notably, early-stage SSB feeding (3 h) failed to induce significant parameter shifts, revealing fundamental differences in temporal response patterns between these phylogenetically distinct herbivores.

### Multidimensional perturbations in metabolic networks induced by herbivores infestation

Both BPH and SSB infestation triggered systemic reorganization of rice metabolic networks across five interconnected physiological domains. Algorithmic derivation from electrophysiological parameters quantified perturbations in (i) water metabolism (IWHC, IWUE, IWHT, IWTR), (ii) nutrient metabolism (UNF, UAF, NAC, NTC, NUE), (iii) leaf metabolic activity (MF, MR, MA), (iv) dielectric substance transport (Kn_R_, Kn_Z_, Kn_XC_, Kn_XL_, ICR_R_, ICF_R_, ICR_Z_, ICF_Z_, ICR_C_, ICF_C_, ICR_L_, ICF_L_), and (v) energy metabolism (ΔG_R-U_, ΔG_R_, ΔG_Z-U_, ΔG_Z_, ΔG_Xc-U_, ΔG_Xc_, ΔG_Xl-U_, ΔG_Xl_) (Fig. 3B-F), revealing herbivore-specific modulation patterns.

For intracellular water metabolism indicators, BPH feeding at 3, 8, and 24 h reduced IWTR to 47.21%, 36.05%, and 32.52% of control levels respectively (*p* < 0.01). SSB_feeding at 8 h decreased IWTR to 28.03% of controls (*p* < 0.01). No significant changes occurred in 3 h (Fig. 3B, Table S4); For intracellular nutrient metabolism indicators, NAC (3 h: 39.56%; 8 h: 43.41%; 24 h: 36.81% of controls) and NTC (3 h: 41.43%; 8 h: 41.94%; 24 h: 38.36% of controls) declined progressively under BPH infestation (*p* < 0.01). SSB_8 h caused acute NAC/NTC reduction to 28.57%/27.37% of controls (*p* < 0.01) (Fig. 3C, Table S5); For leaf metabolic activity indicators, MF decreased to 4.33% (BPH_24 h) and 4.04% (SSB_8 h) of controls (*p* < 0.01). MR and MA exhibited significant reductions across all BPH infestation durations (MR, 3 h: 18.25%; 8 h: 15.71%; 24 h: 10.37%; MA, 3 h: 53.24%; 8 h: 50.23%; 24 h: 43.17%) and in SSB_8 h plants (MR, 7.95%; MA, 38.19%) compared to untreated specimens (*p* < 0.01) (Fig. 3D, Table S6); For intracellular dielectric substance transport indicators, ICR_R_ increased to 233.33%, 273.33%, and 380.00% of control levels and ICR_Z_ increased to 219.57%, 315.22%, and 350.00% of control levels under 3 $\sim$ 24 h under BPH infestation (*p* < 0.01). SSB_8 h increased ICR_R_ and ICR_Z_ to 488.88% and 467.39% of controls (*p* < 0.01) (Fig. 3E, Table S7); For energy metabolism indicators of various intracellular dielectric substances, BPH_3 h and BPH_8 h decreased ΔG_R_ (3 h: 27.55%; 8 h: 40.38%), ΔG_Z_ (3 h: 30.36%; 8 h: 36.91%), ΔG_Xc_ (3 h: 40.55%; 8 h: 64.67%), and ΔG_Xl_ (3 h: 36.61%; 8 h: 61.89%) (*p* < 0.01). SSB_8 h caused broader reductions, ΔG_R-U_ (39.92%), ΔG_R_ (32.98%), ΔG_Z-U_ (36.17%), ΔG_Z_ (29.28%), ΔG_Xc_ (65.51%), and ΔG_Xl-U_ (62.46%) significantly decreased (*p* < 0.01), while SSB_3 h only caused ΔG_R_ (45.00%) and ΔG_Xc_ (64.32%) significant reductions (*p* < 0.01) (Fig. 3F, Table S8).

### Development of agronomic trait prediction models using herbivore-responsive electrophysiological indices

**Fig. 4:**
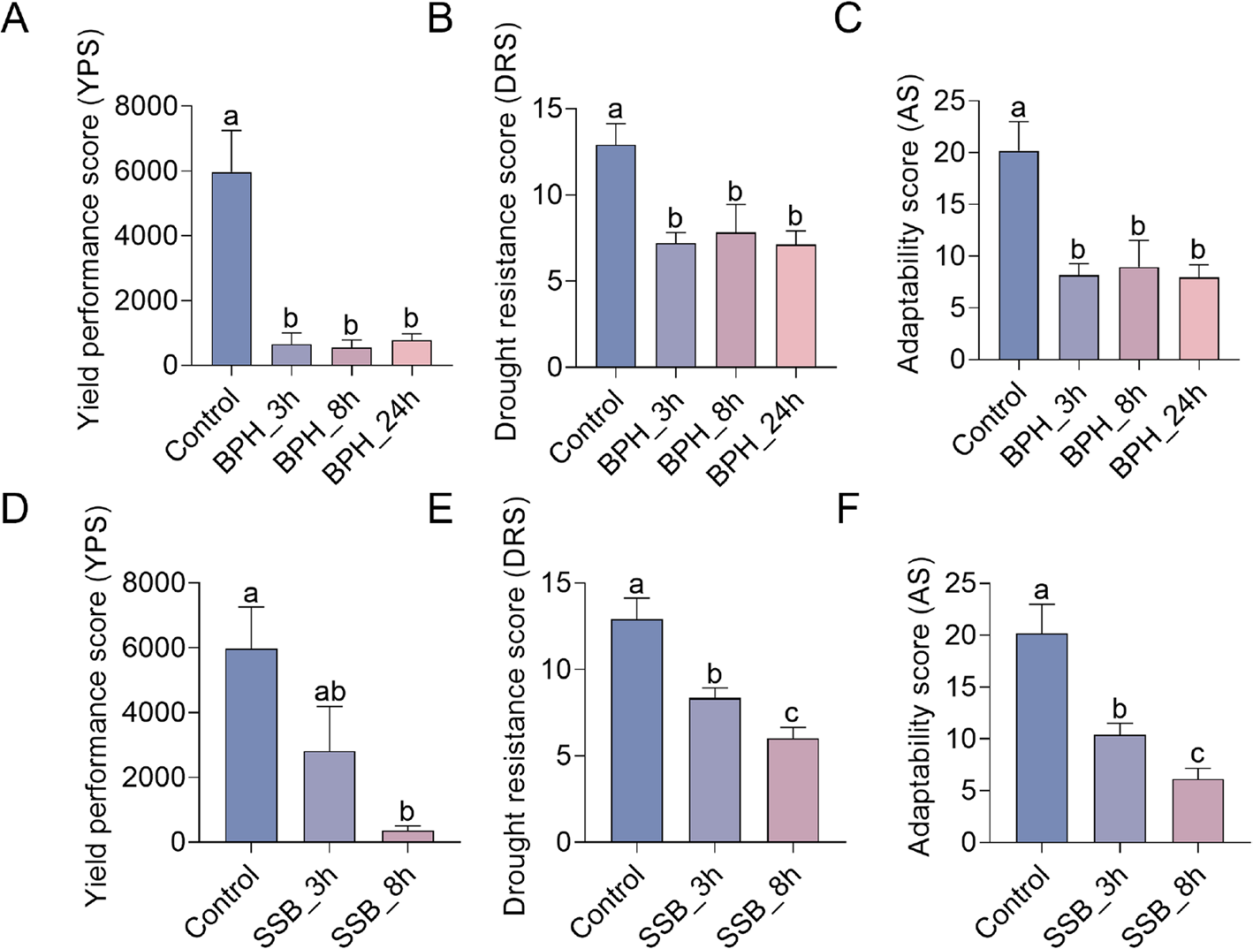
Agronomic trait prediction models based on electrophysiological indicators in response to herbivores infestation. (**A**-**C**) Models of yield performance score (YPS, **A**), drought resistance score (DRS, **B**), and adaptability score (AS,** C**) under BPH infestation at different time points. (**D**-**F**) Models of yield performance score (YPS, **D**), drought resistance score (DRS, **E**), and adaptability score (AS, **F**) under SSB infestation at different time points. Error bars represent ± standard errors. Different letters indicate significant differences among treatments (*p* < 0.05, one-way ANOVA followed by Duncan’s multiple range test)

The multidimensional metabolic perturbations induced by BPH and SSB infestations necessitated the development of quantitative models to assess agronomic impacts through electrophysiologically derived indices. Three quantitative models were established using derived electrophysiological parameters to quantify infestation severity through multidimensional metabolic perturbations. The yield potential score (YPS), integrating intracellular water/nutrient translocation rates, metabolic activity, and leaf specific effective thickness as high-yield determinants, was calculated as $\mathrm{YPS}=\sqrt[4]{\mathrm{IWTR}\times \mathrm{MA}\times \mathrm{d}}$. BPH infestation demonstrated severe YPS suppression to 11% (3 h), 9% (8 h), and 13% (24 h) of control levels. SSB feeding induced progressive impairment, reducing YPS to 47% of controls at 3 h with acute suppression to 6% at 8 h (Fig. 4A, D), reflecting differential temporal impacts of chewing versus phloem-feeding herbivory on yield architecture.

The drought resistance score (DRS) was calculated as $\mathrm{DRS}=\sqrt[4]{\mathrm{IWHT}\times \mathrm{IWHC}\times \mathrm{MA}}$, quantifying rice's drought resistance through intracellular water retention capacity, water holding duration, and metabolic activity. BPH infestation reduced DRS to 56% (3 h), 61% (8 h), and 55% (24 h) of control levels, while SSB feeding decreased DRS to 52% (3 h) and 30% (8 h) of untreated plants (Fig. 4B, E). An adaptability score $\mathrm{AS}=\sqrt[4]{\mathrm{IWHT}\times \mathrm{IWHC}\times \mathrm{MA}\times \mathrm{NTC}}$ further incorporated nutrient transport competence, requiring both robust water homeostasis and active nutrient transport competence to sustain metabolic flexibility. BPH infestations reduced AS to 39% (3 h), 44% (8 h), and 39% (24 h) of control levels, demonstrating progressive adaptive capacity erosion. In contrast, SSB elicited acute AS suppression to 51% (3 h) and 30% (8 h) of untreated plants (Fig. 4C, F), reflecting chewing herbivores' disruptive impact on the synergistic coordination between hydraulic and nutritional systems.

### Coupling between electrophysiological parameters and defense phytohormones

**Fig. 5:**
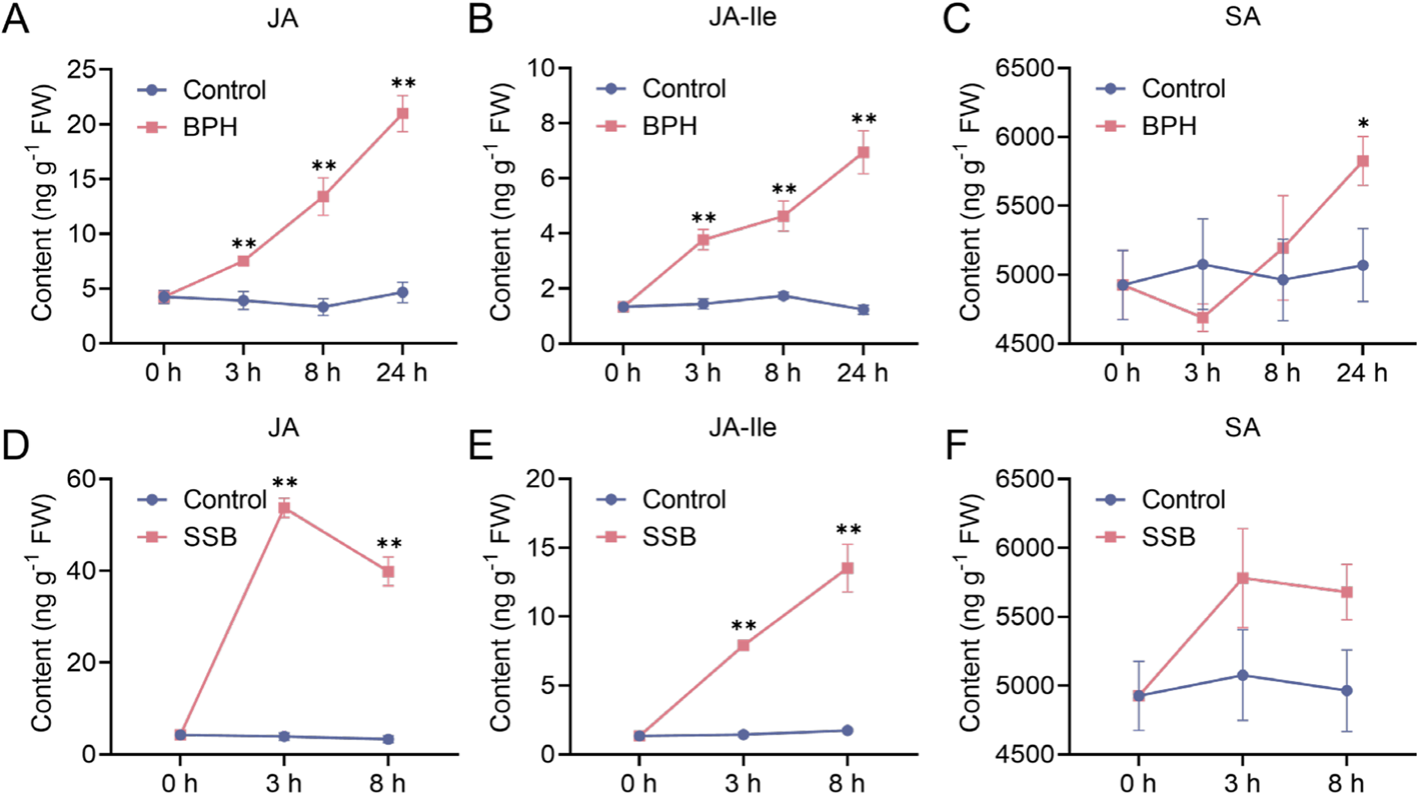
Dynamic changes in rice defense-related phytohormones in response to herbivores infestation. **A**-**C** Contents of JA (**A**), JA-Ile (**B**), and SA (**C**) in rice under BPH feeding at 0 h (h), 3 h, 8 h, and 24 h, respectively. (**D**-**F**) Contents of JA (**D**), JA-Ile (**E**), and SA (**F**) in rice under SSB feeding at 0 h, 3 h, and 8 h, respectively. Asterisks indicate significant differences (** *p* < 0.01; * *p* < 0.05; Student’s t-test)

Phytohormone quantification revealed distinct temporal induction patterns of defense regulators under herbivore attack. Continuous BPH feeding triggered progressive JA accumulation, with JA level increasing 1.77-fold (3 h), 3.15-fold (8 h), and 4.94-fold (24 h) compared to the controls (Fig. 5A). JA-Ile quantification exhibited even stronger amplification, reaching 2.82-fold (3 h), 3.46-fold (8 h), and 5.19-fold (24 h) induction (Fig. 5B). SA showed minimal response to BPH infestation, with only a 1.15-fold increase at 24 h (Fig. 5C). In contrast, SSB infestation elicited rapid JA surges of 12.64-fold (3 h) and 9.38-fold (8 h) (Fig. 5D), accompanied by JA-Ile elevations of 5.92-fold (3 h) and 10.11-fold (8 h) (Fig. 5E), while SA remained unchanged across SSB treatments (Fig. 5F).

**Fig. 6:**
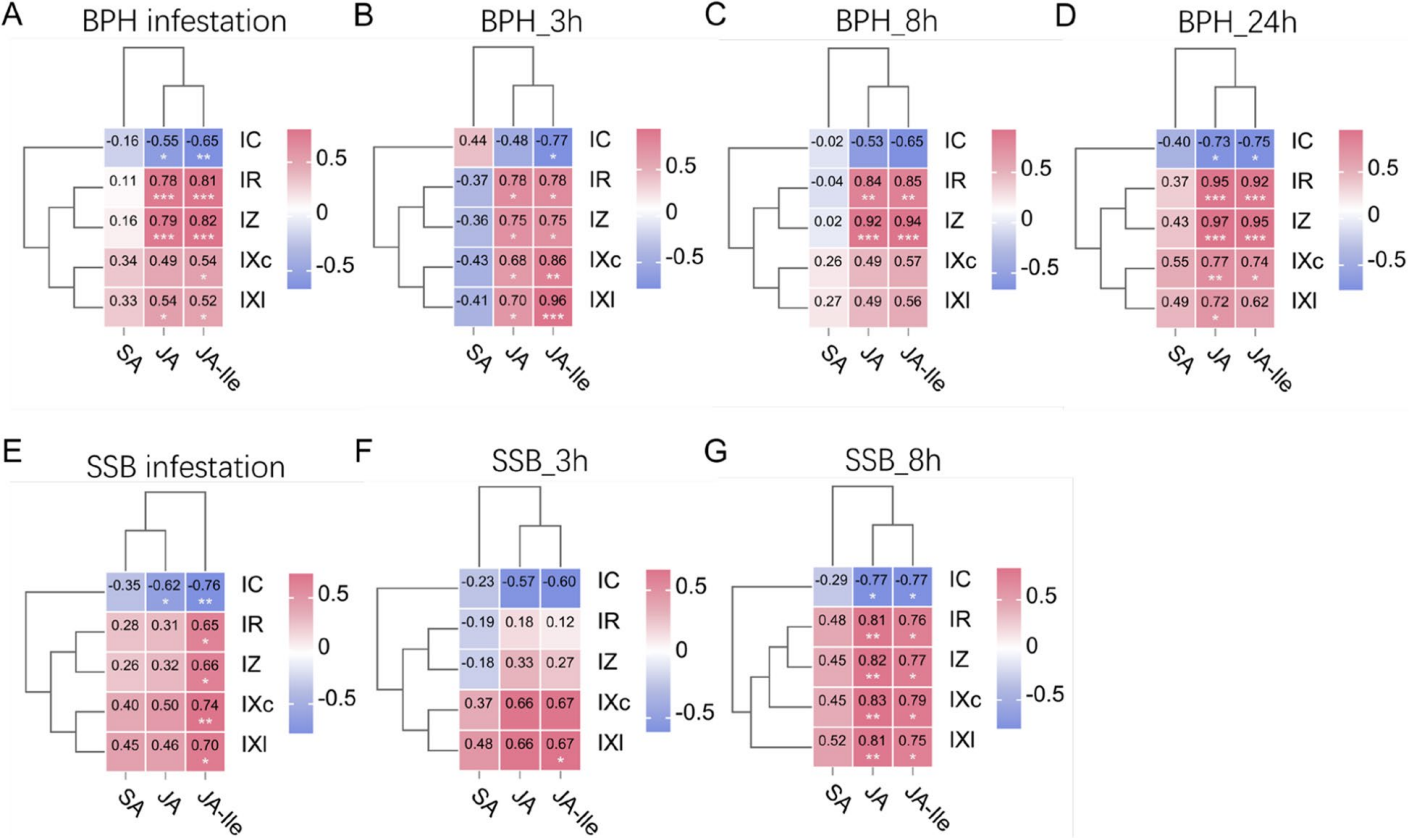
Correlation analysis between rice defense-related phytohormones and electrophysiological parameters under herbivores infestation. (**A**-**D**) Pearson correlation analysis between defense-related phytohormones and electrophysiological parameters in rice during continuous BPH infestation spanning 3–24 h (**A**), with discrete intervals: 3 h (**B**), 8 h (**C**), and 24 h (**D**), respectively. (**E**–**G**) Pearson correlation analysis between defense-related phytohormones and electrophysiological parameters in rice during continuous SSB infestation spanning 3–8 h (**E**), with discrete intervals: 3 h (**F**) and 8 h (**G**), respectively. The numbers in the heatmap represent pearson correlation coefficients, with positive and negative values indicating positive and negative correlations, respectively. Asterisks indicate significant differences (*** *p* < 0.001; ** *p* < 0.01; * *p* < 0.05; Student’s t-test)

Integrated correlation analysis across infestation periods demonstrated strong positive associations between JA/JA-Ile and impedance parameters under BPH stress (JA-IR: *r* = 0.78, *p* < 0.001; JA-IZ: *r* = 0.79, *p* < 0.001; JA-Ile-IR: *r* = 0.81, *p* < 0.001; JA-Ile-IZ: *r* = 0.82, *p* < 0.001), with JA-Ile additionally showing negative correlation to capacitance (JA-Ile-IC: *r* = –0.65, *p* < 0.01) (Fig. 6A). Time-resolved analysis and scatter plots with linear fits revealed dynamic coupling patterns: BPH_3 h specifically linked JA-Ile to reactance parameters (JA-Ile-IXc: *r* = 0.86, *R*^*2*^ = 0.71; JA-Ile-IXl: *r* = 0.96, *R*^*2*^ = 0.92; *p* < 0.01) (Fig. 6B, Fig. S1), whereas later stages (8 h/24 h) showed dominant JA/JA-Ile correlations with IR (JA-IR: *r* = 0.84/0.95, *R*^*2*^ = 0.77/0.88; JA-Ile-IR: *r* = 0.85/0.92, *R*^*2*^ = 0.78/0.82; *p* < 0.01) and IZ (JA-IZ: *r* = 0.92/0.97, *R*^*2*^ = 0.89/0.91; JA-Ile-IZ: *r* = 0.94/0.95, *R*^*2*^ = 0.87/0.88; *p* < 0.001) (Fig. 6C, D, Fig. S2, S3). SSB infestation exhibited contrasting relationships, with pooled 3 ~ 8 h data showing JA-Ile associations to IXc (*r* = 0.74; *p* < 0.01) and IC (*r* = −0.76; *p* < 0.01) (Fig. 6E). SSB_8 h specifically strengthened JA correlations across multiple parameters (JA-IR: *r* = 0.81, *R*^*2*^ = 0.76; JA-IZ: *r* = 0.82,* R*^*2*^ = 0.70; JA-IXc: *r* = 0.83, *R*^*2*^ = 0.76; IXl: *r* = 0.81, *R*^*2*^ = 0.71; *p* < 0.01) (Fig. 6G, Fig. S5), while SSB_3 h showed no significant hormone-parameter linkages (Fig. 6F, Fig. S4).

### Overexpression of salivary elicitors in rice mimics feeding and regulates energy reallocation between plant growth and defense

**Fig. 7:**
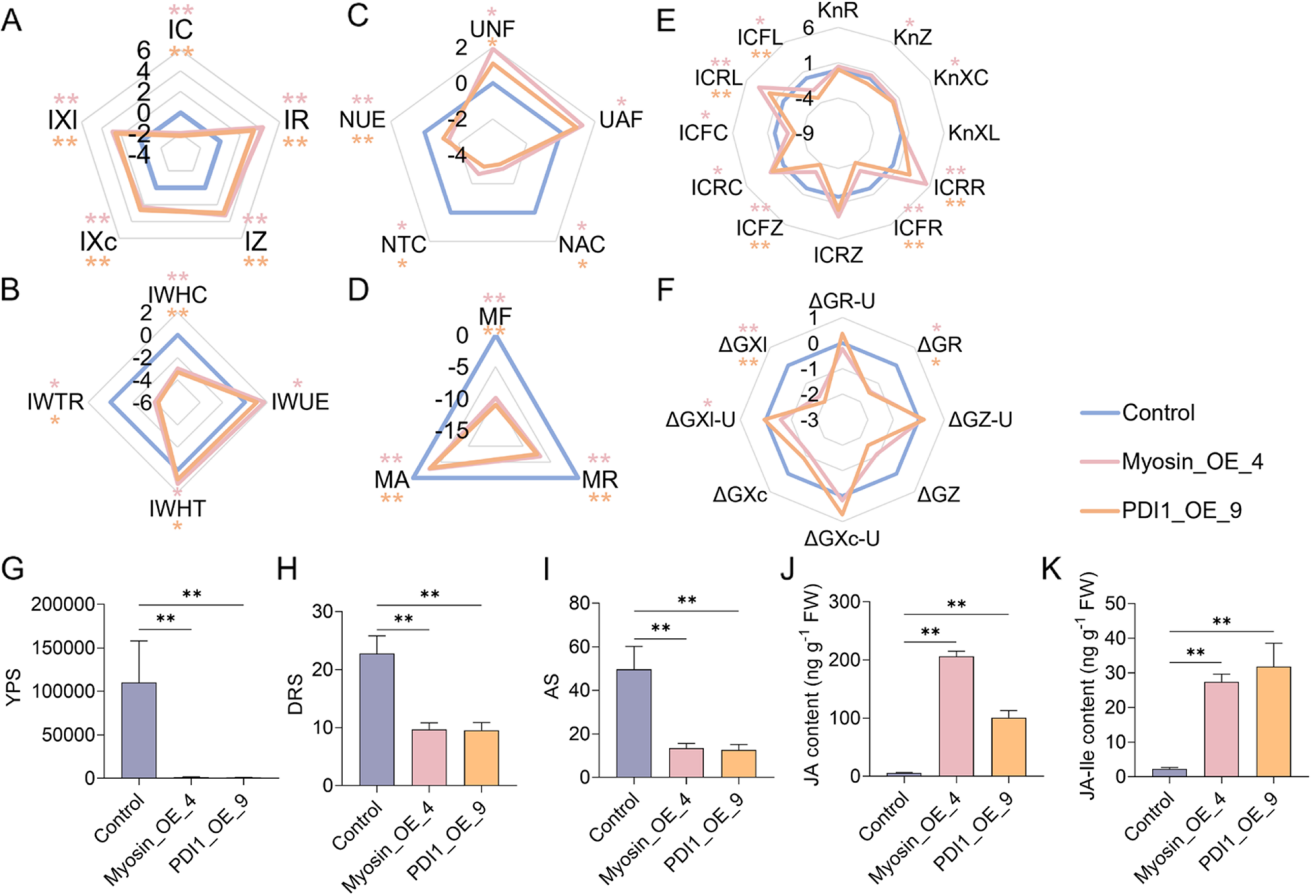
Transgenic rice overexpressing planthopper-derived elicitors mimics BPH feeding and regulates energy reallocation. (**A**-**F**) Electrophysiological parameters (**A**), intracellular water metabolism indicators (**B**), intracellular nutrient metabolism indicators (**C**), leaf metabolic activity indicators (**D**), intracellular dielectric substance transport indicators (**E**), and energy metabolism indicators of various intracellular dielectric substances (**F**) in wild-type (Control), myosin-overexpressed (Myosin_OE_4), and PDI1-overexpressed (PDI1_OE_9) rice plants, respectively. Treatment group values expressed as log_2_-fold changes relative to the control group, respectively. (**G**-**I**) Models of yield performance score (YPS, **G**), drought resistance score (DRS, **H**), and adaptability score (AS,** I**) in Control, Myosin_OE_4, and PDI1_OE_9 groups, respectively. (**J**, **K**) Contents of JA (**J**) and JA-Ile (**K**) in Control, Myosin_OE_4, and PDI1_OE_9 groups, respectively. Asterisks indicate significant differences (** *p* < 0.01; * *p* < 0.05; ns, not significant; Student’s t-test)

Building upon previous identifications of planthopper salivary elicitors: Myosin from SBPH [45] and PDI1 from BPH [46], we investigated their roles in systemic resistance by generating rice overexpression lines, namely Myosin_OE_4 and PDI1_OE_9. qRT-PCR analysis confirmed the successful construction of transgenic plants, with both the Myosin_OE_4 and PDI1_OE_9 lines exhibiting normal growth at the 50-day stage (Fig. S6). Compared to control plants, Myosin_OE_4 exhibited pronounced electrophysiological reprogramming, with IC reduced to 25.36% of control levels and substantial increases in IR (19.90-fold), IZ (9.47-fold), IXc (4.97-fold), and IXl (7.23-fold) (Fig. 7A). Similarly, PDI1_OE_9 showed comparable alterations, with IC reduced to 21.39% of the control and increases in IR (10.47-fold), IZ (8.17-fold), IXc (6.03-fold), and IXl (5.86-fold) (Fig. 7A). These findings suggest that overexpression of Myosin and PDI1 influences active intracellular water and nutrient transport, as well as broader metabolic activity in rice leaves. In Myosin_OE_4, water metabolism indicators IWTR and IWHC decreased to 6.44% and 12.74% of control values, respectively (Fig. 7B). NTC and NAC declined to 15.78% and 12.36% of the control, respectively (Fig. 7C). MF, MR, and MA reached 0.10%, 0.84%, and 12.70% of control levels, respectively (Fig. 7D). The PDI1_OE_9 line showed similar trends: IWTR and IWHC dropped to 5.15% and 10.04% of the control (Fig. 7B), NTC and NAC to 11.18% and 9.99% (Fig. 7C), and MF, MR, and MA to 0.05%, 0.56%, and 11.19% (Fig. 7D), respectively. Dielectric substance transport parameters exhibited proportional impairments. In Myosin_OE_4 and PDI1_OE_9, resistive (ICR_R_: 43.65-fold and 6.68-fold), impedance (ICR_Z_: 7.00-fold and 3.73-fold), capacitive (ICR_C_: 4.30-fold and 3.46-fold), and inductive (ICR_L_: 16.08-fold and 4.92-fold) transport resistances were significantly elevated compared to controls (Fig. 7E). Indicators of energy metabolism also declined: ΔG_R_ and ΔG_Xl_ in Myosin_OE_4 reached 36.92% and 30.58% of control values, respectively, and 35.15% and 24.80% in PDI1_OE_9 (Fig. 7F). These physiological disturbances resulted in agronomic impairments. In Myosin_OE_4 and PDI1_OE_9, YPS dropped to 1.12% and 0.83%, DRS to 42.61% and 41.96%, and AS to 26.98% and 25.49% of control levels, respectively (Figs. 7G–I). Detailed data are provided in Table S9. Furthermore, defense-related hormonal levels were markedly elevated. JA and JA-Ile increased 29.03-fold and 10.05-fold in Myosin_OE_4, and 17.00-fold and 14.01-fold in PDI1_OE_9 compared to controls (Fig. 7J, K).

## Discussion

Electrophysiological signals represent one of the fastest mechanisms by which plants respond to environmental changes. These signals are closely associated with cellular water potential, ion concentration, membrane permeability, and turgor pressure [54, 55]. Previous studies have shown that under drought or salt stress, plant leaf cells exhibit significant changes in electrical parameters such as IR, IZ, and IXc, reflecting the restructuring of cell membrane functionality [38, 56, 57]. These electrical parameters serve as rapid, non-invasive indicators of plant physiological states. Capacitance changes often correlate with cell expansion and contraction, while resistance and impedance more accurately reflect the movement of water and ions, indirectly indicating dynamic changes in membrane permeability and tissue structure [58]. These shifts frequently precede visible growth inhibition and tissue necrosis, underscoring their timeliness and sensitivity. With the advancement of electrophysiological measurement technologies, more research has explored their application in biotic stress scenarios such as pathogen infection [42] and herbivore infestation [41], with growing interest in their potential for early detection of plant defense responses. However, studies investigating the electrophysiological responses of rice to herbivores and their mechanistic links with defense-related phytohormones remain limited, particularly in terms of systematic correlation analysis.

In this study, we systematically compared the dynamic effects of two types of herbivorous pests (BPH and SSB) on rice electrophysiological parameters, which serve as real-time reporters of underlying physiological and defense states. Sustained feeding by the phloem-feeding BPH progressively enhanced IR and IZ (Fig. 3A)—electrophysiological signatures indicative of defense-induced membrane fortification (callose deposition and lignin synthesis at feeding site) and reduced ion permeability, a common response to limit apoplastic solute loss during piercing-sucking herbivory [26, 59, 60]. In contrast, the tissue-chewing SSB elicited a delayed but broader-spectrum response, significantly elevating IR, IZ, IXc, and IXl after 8 h (Fig. 3A). This coordinated shift reflects systemic breakdown of cellular compartmentalization due to mechanical damage, leading to disrupted electrochemical gradients essential for nutrient uptake and turgor maintenance. The involvement of IXc and IXl specifically points to alterations in membrane capacitance and inductive properties, likely tied to calcium flux oscillations (IXc) and proton pump activity (IXl) that gate early defense signaling [61]. These herbivore-specific temporal dynamics in electrophysiological traits directly quantify perturbations in intracellular water, nutrient, and dielectric material transport capacities. Regarding intracellular water metabolism, both BPH and SSB significantly reduced IWTR (Fig. 3B), suggesting that herbivore infestation impairs water transport, slowing growth and development. This may correlate with impaired aquaporin function and osmotic homeostasis, a conserved response to herbivore-triggered hydraulic failure [21]. Nutrient metabolism indicators such as NAC and NTC were also significantly decreased (Fig. 3C), indicating that herbivore infestation reduced the plant's active nutrient transport and metabolic capacity, mirroring repression of amino acid and sucrose transporters to limit nutrient availability for pests [62]. These reductions reflect a diminished ability of rice to acquire and utilize nutrient resources. The decrease in MR (Fig. 3D) further implies a decline in the movement of reactive substances and metabolic vigor, while MA, derived from MF and MR, indicates a general weakening of life activity. In terms of dielectric material transport, decreases in ICR_R_ and ICR_Z_ (Fig. 3E) suggest increased resistance to dielectric flow and reduced membrane transport efficiency, possibly due to increased membrane lipid content or redistribution of charged metabolites (defense-related secondary compounds) across membranes [63]. In the energy metabolism of dielectric materials, both BPH and SSB feeding (3 h or 8 h) leading to reductions in the energy required for the metabolism of resistive, capacitive, and reactive cellular materials (Fig. 3F), reflecting decreased cellular metabolic capacity. Decrease of metabolic energy flux quantifying the energetic cost of defense activation, including resource diversion from growth to JA biosynthesis and trypsin inhibitor production [64]. Notably, this suppression was partially reversed after 24 h of BPH feeding, while the 8-h SSB treatment showed significantly stronger effects than the 3-h treatment, likely due to the more severe tissue damage caused by chewing. In contrast, BPH’s piercing-sucking mouthparts cause minimal physical damage, allowing the plant to activate a range of defense responses [65]. Overall, BPH-induced changes in electrophysiological parameters became progressively more pronounced with longer feeding durations. For SSB treatment, early feeding had limited electrophysiological impact, but in the later stage, nearly all major parameters fluctuated dramatically. These contrasting responses reflect the fundamentally different feeding strategies of the two herbivores.

To further elucidate rice defense mechanisms against herbivores attack, we conducted pearson correlation analyses between defense phytohormones and electrophysiological parameters to assess the potential of electrical signals as surrogate indicators of resistance (Fig. 5, 6). Previous studies in Arabidopsis have demonstrated the correlation between plant electrical signals and plant hormones: electrical signals trigger changes in intracellular calcium concentration, and calcium influx mediated by GLR activates the JA biosynthesizing enzyme LOX6, which in turn activates JA synthesis [61]. Our results revealed species-specific coupling patterns between hormones and electrical parameters, with significant differences in timing and intensity. During BPH infestation (3 ~ 24 h), JA and JA-Ile accumulation showed time-dependent increases and were strongly positively correlated with IR and IZ (Fig. 6A). These parameters represent the resistance to resistive and dielectric substance flow in cells and can be interpreted as feedback on membrane permeability and charge transport capacity [66]. Upon stress, electrical signals propagate rapidly via plasmodesmata, redistributing water and solutes across membranes and activating both local and systemic defense responses [39]. Previous studies have shown that JA signaling is tightly associated with ionic fluxes, particularly Ca^2^⁺ and K⁺ movement, as well as membrane polarization through H⁺-ATPases activation [41, 67]. These ion-related activities contribute to early pattern-triggered immunity and leave quantifiable electrical signatures—namely changes in resistance, impedance, and reactance—which were observed to correlate with JA/JA-Ile dynamics in our study [68]. Thus, the strong alignment between JA signaling and electrophysiological traits is not merely correlational but reflects known mechanistic underpinnings of plant immune activation. The early correlation between JA-Ile and IXc/IXl likely reflects the plant’s initial bioelectrical response to herbivore recognition, occurring prior to large-scale ionic mobilization. As measures of capacitive and inductive reactance, IXc and IXl characterize frequency-dependent impedance properties of the cell membrane system. In this context, increased IXc may indicate early alterations in membrane dielectric properties (e.g., lipid bilayer rearrangement or transient dipole alignment), while IXl may reflect delayed current responses arising from structural or functional inductive elements—such as gating kinetics of ion channels or conformational shifts in membrane proteins [61]. These rapid but localized perturbations act as low-energy signal precursors, preceding macroscopic changes in IR and IZ, and may represent the earliest electrical footprint of JA-Ile-associated pattern recognition signaling. As the infestation progressed, broader hormonal synthesis and resource mobilization likely led to a transition to IR and IZ-dominated electrical responses. Notably, SA levels increased only slightly at 24 h under BPH treatment, without significant correlation to any electrical parameter (Fig. 5C, 6A), suggesting that SA may not play a primary defensive role in this context, or its function is more indirect (e.g., inducing volatiles or PR genes), without directly altering membrane function or ion homeostasis in detectable ways. In contrast, the SSB group showed a different response pattern. Although JA and JA-Ile levels significantly increased at 3 h, no significant correlations with electrical parameters were observed (Fig. 6E), and changes in those parameters remained modest. This suggests that while hormone biosynthesis was initiated, electrical responses lagged behind. Severe tissue damage by SSB may disrupt systemic electrical signaling, temporarily uncoupling hormone accumulation from electrophysiological changes. Additionally, because SSB prefers to bore into stem bases, it may take time for the damage to reach a threshold that elicits systemic electrical changes. By 8 h, however, IR, IZ, IXc, and IXl became highly correlated with JA, marking a transition from early silence to widespread electrophysiological activation (Fig. 6G). This reflects the plant’s shift to an integrated defense response involving water, ions, energy, and signaling. Taken together, during BPH infestation, electrical parameters—especially IR and IZ—closely mirror JA signaling dynamics and may serve as electrophysiological markers of its signaling activity. Early correlations between JA-Ile and IXc/IXl also suggest these parameters could serve as "early warning signals" before hormone upregulation. In contrast, SSB-treated group exhibited a “hormone-first, electrical-later” pattern, indicating that insect feeding mode affects the timing and sequence of signal activation. While electrical parameters cannot fully replace hormonal measurements, their strong correlations with hormone levels support their potential as rapid, non-invasive indicators of herbivore stress, offering a valuable tool for field diagnostics.

Interestingly, the transgenic rice line Myosin_OE_4 provides a compelling example of a defense‑prioritized phenotype triggered by the overexpression of a pest‑derived elicitor. Although Myosin was initially identified as a salivary component of the SBPH, phylogenetic analysis confirmed a high degree of homology between Myosin isoforms from SBPH and BPH, supporting its functional relevance in the BPH–rice interaction model used here (Fig. S7). Functionally, Myosin acts as a classical pathogen‑associated molecular pattern (PAMP)-like elicitor, which is recognized by plant pattern recognition receptors and activates downstream immune signaling via the OsBAK1 co-receptor [45]. Myosin triggers a rapid influx of Ca^2^⁺, ROS bursts, and MAPK phosphorylation (e.g., MPK3/6), all hallmark events of PAMP-triggered immunity [45]. Protein disulfide isomerase 1 (PDI1), on the other hand, represents a redox-active elicitor secreted by BPH. Recent findings have shown that PDI1 not only enhances plant resistance by promoting H₂O₂, JA, and JA-Ile accumulation, but also directly targets and destabilizes the host’s UDP-glucose epimerase 2 (OsUGE2), a negative regulator of rice immunity. The PDI1–OsUGE2 interaction reduces OsUGE2 protein levels via proteasome-mediated degradation, disrupting sugar metabolism and amplifying defense-associated ROS and JA signals [46]. In both Myosin_OE_4 and PDI1_OE_9 transgenic lines, we observed highly consistent electrophysiological profiles indicative of defense activation, including reduced intracellular conductivity (IC) and elevated resistance and impedance values (IR, IZ, IXc, IXl), alongside suppressed physiological metrics of transport and metabolism (e.g., IWTR, NTC, MF), and dramatically increased JA and JA-Ile concentrations (Fig. 7A–D, J–K). These data confirm that both elicitors rewire host metabolism toward a defense-primed state even in the absence of herbivore. Importantly, we leveraged detailed electrophysiological profiling to simulate and quantify these elicitor-induced defense states. The transgenic lines Myosin_OE_4 and PDI1_OE_9 recapitulated herbivory-like electrical signatures—including approximately tenfold elevated IR/IZ and suppressed IC (25% of Control) (Fig. 7A)—demonstrating that parameters such as IC, IR, IZ, IXc, and IXl reliably reflect the shift to a defense-primed physiology. This electrophysiological signature offers a rapid, non-invasive readout to screen and prioritize candidate insect-derived elicitors before extensive bioassays—accelerating the identification of novel resistance inducers. Together, these results confirm that distinct herbivore-derived elicitors can converge on common defense signaling axes in rice, and that electrical parameter profiling provides a powerful platform for the preliminary screening and functional validation of novel elicitors.

In conclusion, this study highlights the potential of electrophysiological traits as a rapid, non-invasive biomarker for decoding rice defense states under herbivore stress. By capturing real-time electrical changes linked to water transport, nutrient flux, and hormonal signaling, we provide compelling evidence that these signals reflect both physiological disruptions and immune activation. The successful replication of herbivore-like electrical profiles in elicitor-overexpressing transgenic lines further underscores the ability of electrophysiological monitoring to simulate defense responses triggered by elicitors, closely resembling those induced by herbivore feeding. This approach provides a powerful tool for the functional screening of elicitors, facilitating more efficient identification of candidates for enhancing plant resistance. These findings open new avenues for applying bioelectrical monitoring in precision agriculture, accelerating the selection of resistant cultivars.

## Supplementary Information

Supplementary Material 1.

## Data Availability

All data generated or analysed during this study are included in this published article and its supplementary information files.

## Abbreviations

BPH: Brown planthopper, *Nilaparvata lugens*
SSB: Striped stem borer, *Chilo suppressalis*
SBPH: Small brown planthopper, *Laodelphax striatellus*
JA: Jasmonic acid
JA-Ile: Jasmonic acid-isoleucine
SA: Salicylic acid
C: Physiological capacitance
R: Physiological resistance
Z: Physiological impedance
Xc: Physiological capacitive reactance
Xl: Physiological inductive reactance
IC: Intrinsic physiological capacitance
IR: Intrinsic physiological resistance
IZ: Intrinsic physiological impedance
IXc: Intrinsic physiological capacitive reactance
IXl: Intrinsic physiological inductive reactance
d: Specific effective thickness
IWHC: Intracellular water holding capacity
IWUE: Intracellular water use efficiency
IWHT: Intracellular water holding time
IWTR: Intracellular water transfer rate
UNF: Unit nutrient flux
UAF: Unit active flux
NAC: Nutrient active transport capacity
NTC: Nutrient transport capacity
NUE: Nutrient use efficiency
MF: Metabolic flux
MR: Metabolic rate
MA: Metabolic activity
Kn_R_: Transfer number of dielectric substance in response to physiological resistance
Kn_Z_: Transfer number of dielectric substance in response to physiological impedance
Kn_XC_: Transfer number of dielectric substance in response to physiological capacitive reactance
Kn_XL_: Transfer number of dielectric substance in response to physiological inductive reactance
ICR_R_: Intracellular conductive resistance of resistive substance
ICF_R_: Intracellular conductive force of resistive substance
ICR_Z_: Intracellular conductive resistance of total dielectric substance
ICF_Z_: Intracellular conductive force of total dielectric substance
ICR_C_: Intracellular conductive resistance of capacitive substance
ICF_C_: Intracellular conductive force of capacitive substance
ICR_L_: Intracellular conductive resistance of inductive substance
ICF_L_: Intracellular conductive force of inductive substance
ΔG_R__-U_: Unit metabolic energy based on resistance
ΔG_R_: Metabolic energy based on resistance
ΔG_Z__-U_: Unit metabolic energy based on impedance
ΔG_Z_: Metabolic energy based on impedance
ΔG_Xc__-U_: Unit metabolic energy based on capacitive reactance
ΔG_Xc_: Metabolic energy based on capacitive reactance
ΔG_Xl__-U_: Unit metabolic energy based on inductive reactance
ΔG_Xl_: Metabolic energy based on inductive reactance
YPS: Yield potential score
DRS: Drought resistance score
AS: Adaptability score
*R*^2^: Multiple correlation coefficient
*r*: Pearson's correlation coefficient
