# Supplementary material for: Electrophysiological signatures decode herbivore-specific defense dynamics and elicitor-induced immune activation in rice
Source: Crop Health. 2025 Sep 30;3(1):19. doi: 10.1007/s44297-025-00058-z (PMC12825979; doi:10.1007/s44297-025-00058-z)
Supplement: Supplementary file 1 — Supplementary Material 1. [file 44297_2025_58_MOESM1_ESM.docx]

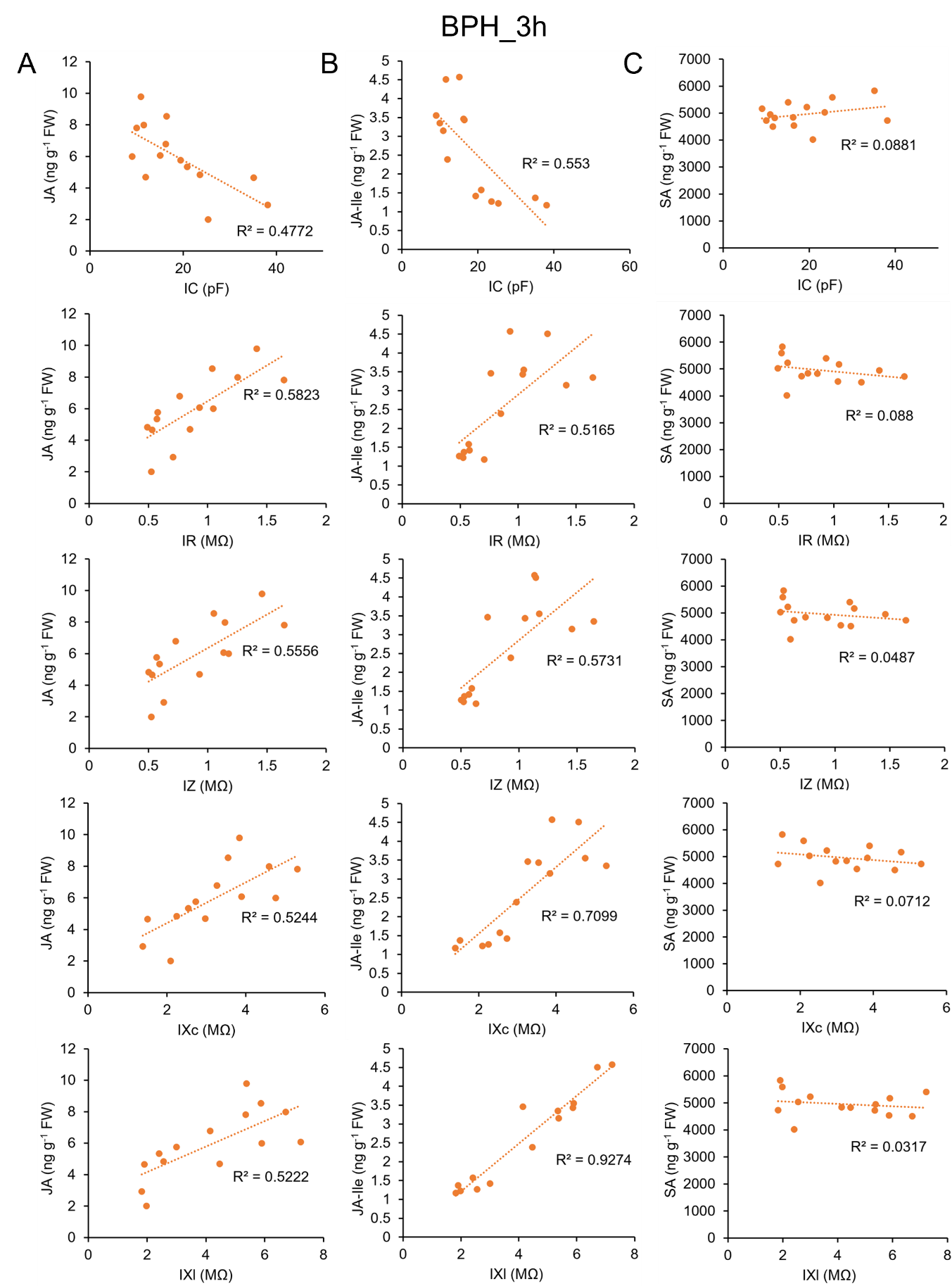
**Electrophysiological signatures decode herbivore-specific defense dynamics and elicitor-induced immune activation in rice**

**Fig. S1** Scatter plots with linear fits showing relationships between electrophysiological parameters and phytohormone contents in Control and BPH_3h groups.

(A–C) Scatter plots with linear fits depicting associations between electrophysiological parameters (IC, IR, IZ, IXc, and IXl) and plant defense hormones: JA (A), JA-Ile (B), and SA (C). Each point represents an individual replicate, with hormone content (Y-axis) plotted against the corresponding electrophysiological parameter value (X-axis). R²: coefficient of determination.


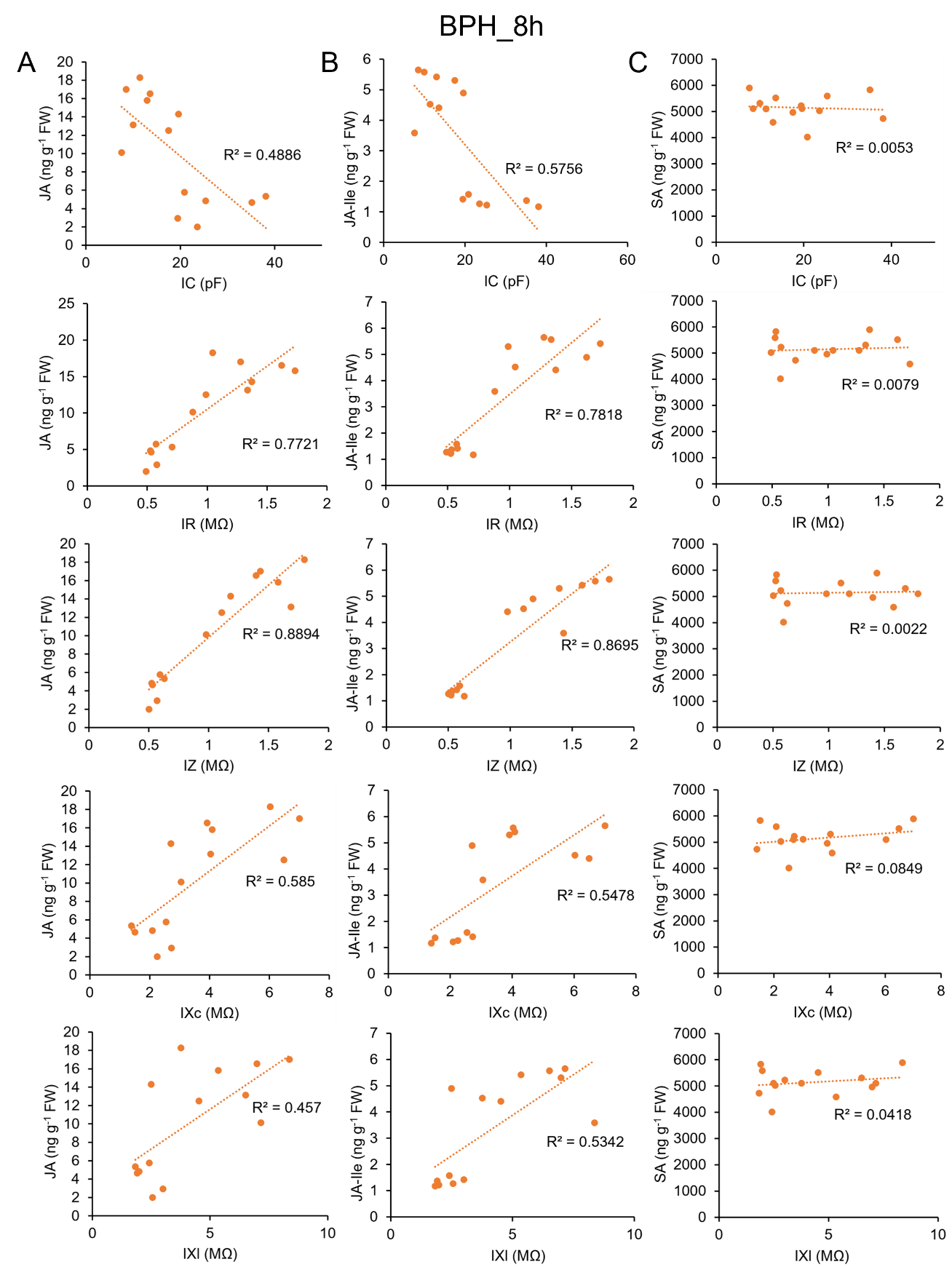


**Fig. S2** Scatter plots with linear fits showing relationships between electrophysiological parameters and phytohormone contents in Control and BPH_8h groups.

(A–C) Scatter plots with linear fits depicting associations between electrophysiological parameters (IC, IR, IZ, IXc, and IXl) and plant defense hormones: JA (A), JA-Ile (B), and SA (C). Each point represents an individual replicate, with hormone content (Y-axis) plotted against the corresponding electrophysiological parameter value (X-axis). R²: coefficient of determination.

**
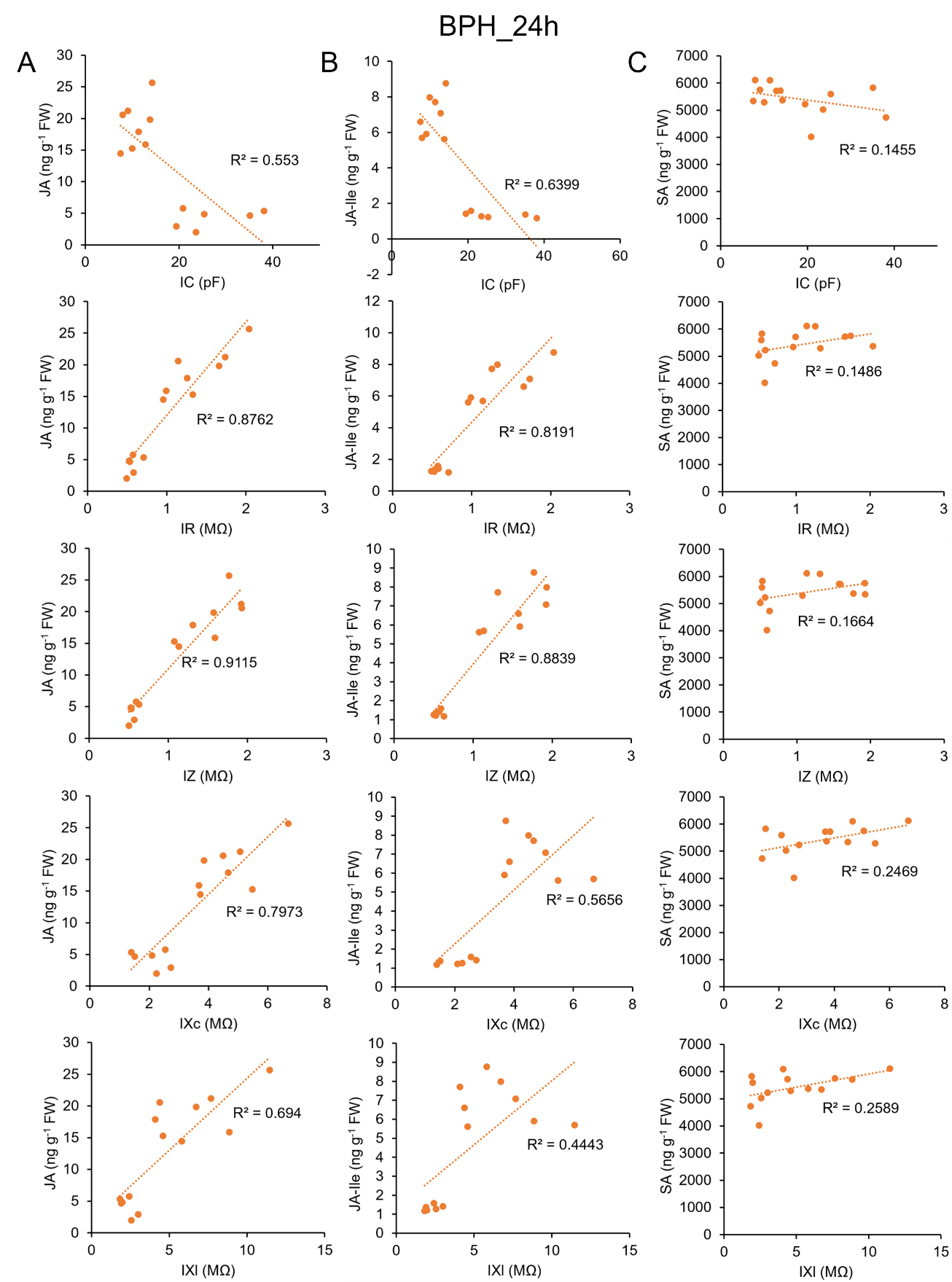
**

**Fig. S3** Scatter plots with linear fits showing relationships between electrophysiological parameters and phytohormone contents in Control and BPH_24h groups.

(A–C) Scatter plots with linear fits depicting associations between electrophysiological parameters (IC, IR, IZ, IXc, and IXl) and plant defense hormones: JA (A), JA-Ile (B), and SA (C). Each point represents an individual replicate, with hormone content (Y-axis) plotted against the corresponding electrophysiological parameter value (X-axis). R²: coefficient of determination.

**
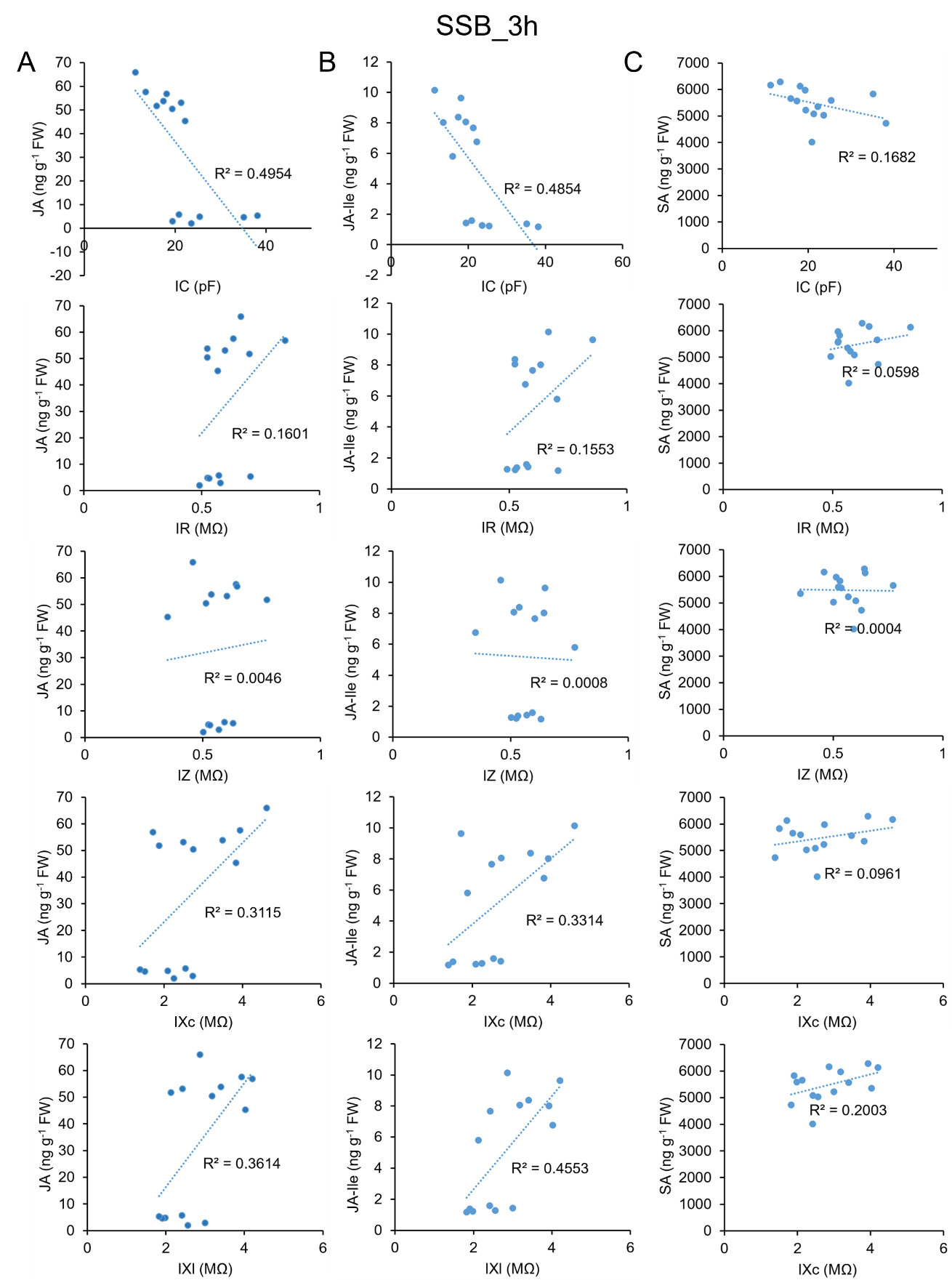
**

**Fig. S4** Scatter plots with linear fits showing relationships between electrophysiological parameters and phytohormone contents in Control and SSB_3h groups.

(A–C) Scatter plots with linear fits depicting associations between electrophysiological parameters (IC, IR, IZ, IXc, and IXl) and plant defense hormones: JA (A), JA-Ile (B), and SA (C). Each point represents an individual replicate, with hormone content (Y-axis) plotted against the corresponding electrophysiological parameter value (X-axis). R²: coefficient of determination.

**
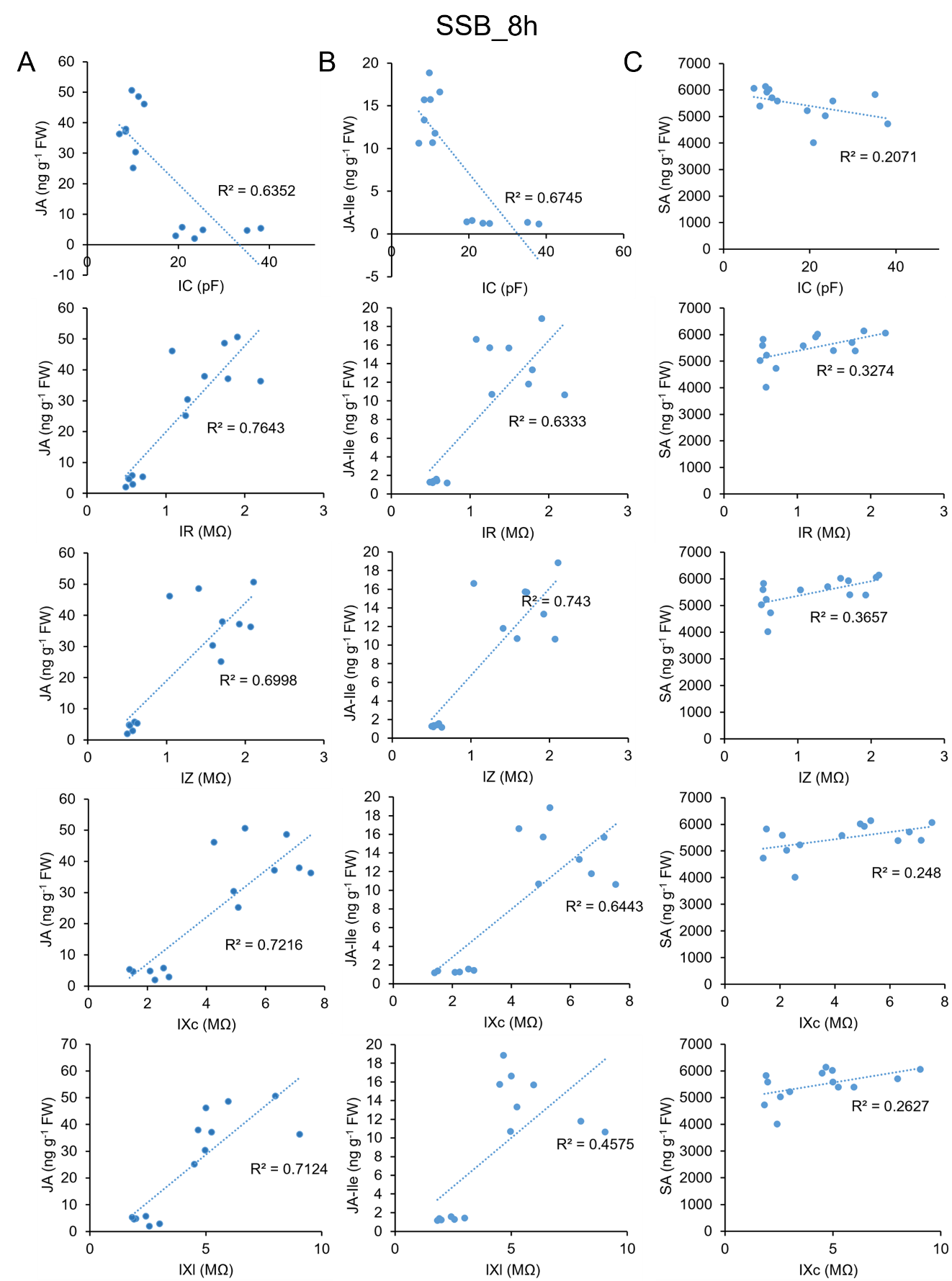
**

**Fig. S5** Scatter plots with linear fits showing relationships between electrophysiological parameters and phytohormone contents in Control and SSB_8h groups.

(A–C) Scatter plots with linear fits depicting associations between electrophysiological parameters (IC, IR, IZ, IXc, and IXl) and plant defense hormones: JA (A), JA-Ile (B), and SA (C). Each point represents an individual replicate, with hormone content (Y-axis) plotted against the corresponding electrophysiological parameter value (X-axis). R²: coefficient of determination.

**
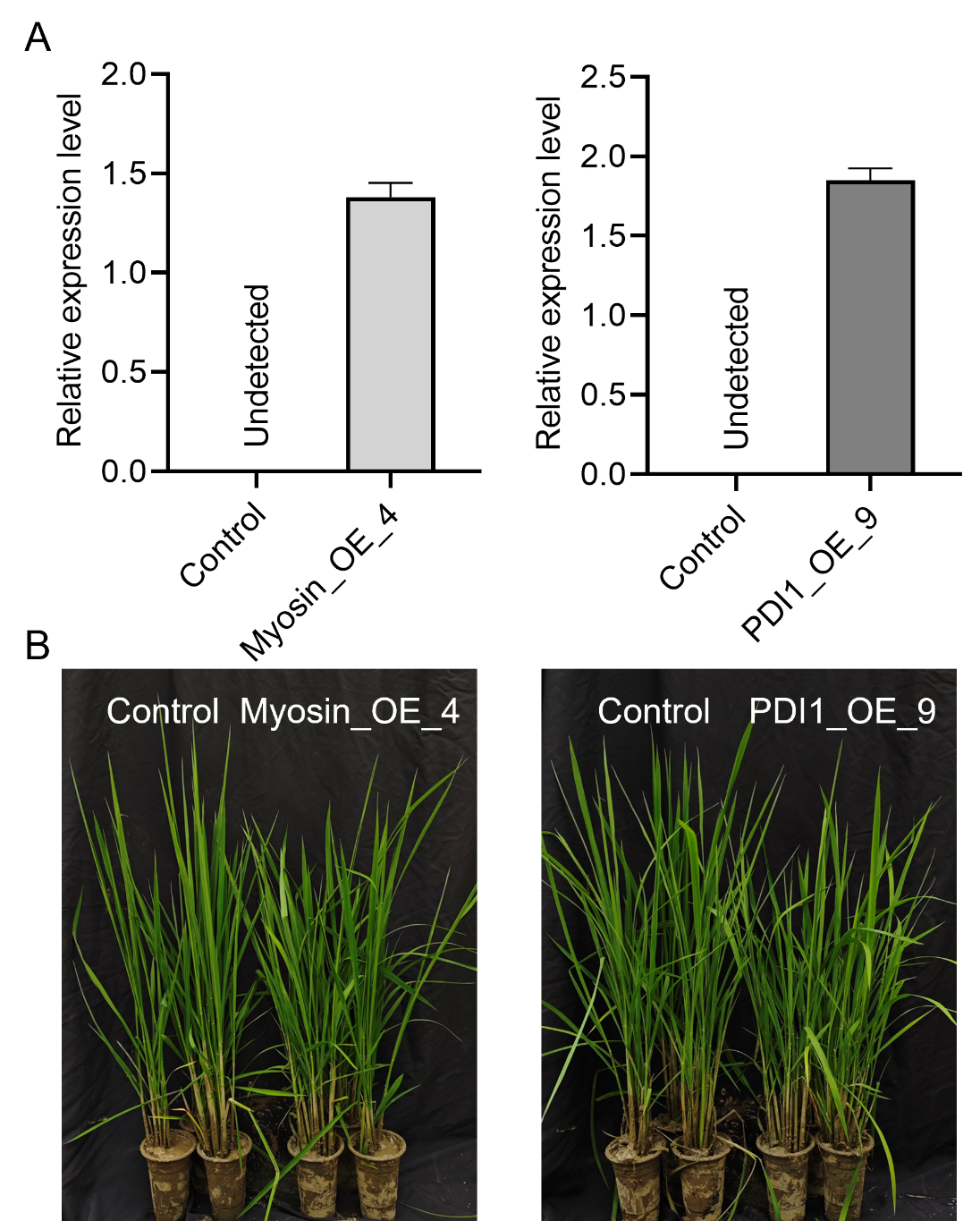
**

**Fig. S6** Expression of Myosin and PDI1 and growth status of Myosin/PDI1-expressing rice lines

(A) Mean expression levels (+ SE, *n* = 3) of the *Myosin* gene in Control and Myosin_OE_4 rice (left panel), and the *PDI1* gene in PDI1_OE_9 rice (right panel). (B) Typical images of the growth status of Control, Myosin_OE_4, and PDI1_OE_9 rice seeds 50 days after germination.


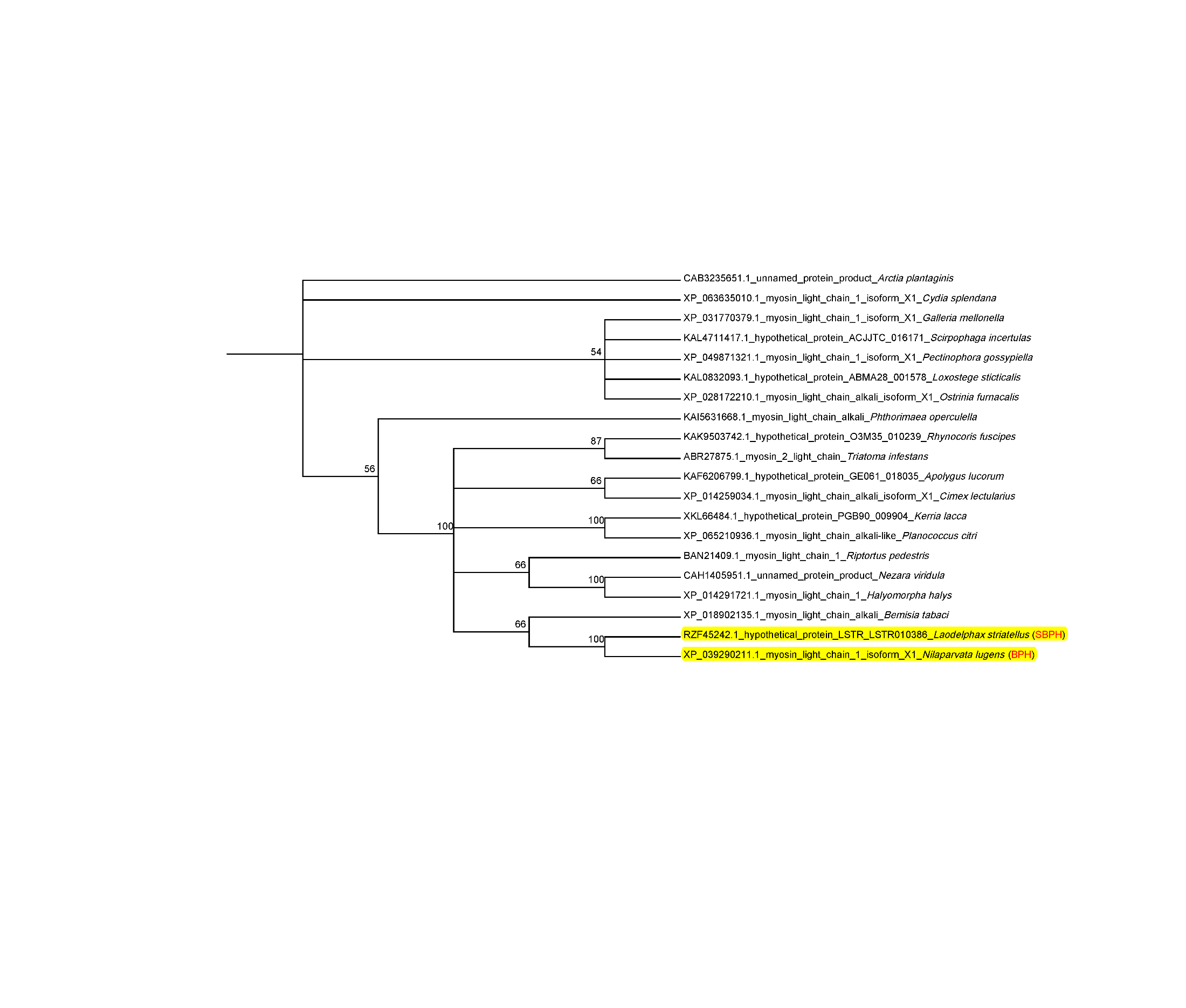


**Fig. S7** Phylogenetic analysis of the top 20 insect proteins with highest similarity to SBPH salivary myosin protein

Branches highlighted in yellow represent myosin proteins from SBPH and BPH salivary, respectively. The Cluster W program in MEGA software was used for aligning the protein sequences and constructing an evolutionary tree using the Neighbor Joining algorithm (Poisson model assessed through 1,000 bootstrap tests).

**Table S1** The equations and parameters of the model of the leaves C with the clamping force (F)

| Sample | x_0_ | h | Equation |
| --- | --- | --- | --- |
| Control | 21.37 | 58.70 | C=21.37+58.70 F  *R*^2^=0.99, *p* < 0.01, *n*=63 |
| BPH_3h | 12.61 | 31.10 | C=12.61+31.10 F  *R*^2^=0.98, *p* < 0.01, *n*=63 |
| BPH_8h | 13.38 | 39.00 | C=13.38+39.00 F  *R*^2^=0.96, *p* < 0.01, *n*=63 |
| BPH_24h | 11.84 | 77.80 | C=11.84+77.80 F  *R*^2^=0.97, *p* < 0.01, *n*=63 |
| Control | 21.37 | 58.70 | C=21.37+58.70 F  *R*^2^=0.98, *p* < 0.01, *n*=63 |
| SSB_3h | 18.03 | 47.00 | C=18.03+47.00 F  *R*^2^=0.99, *p* < 0.01, *n*=63 |
| SSB_8h | 9.31 | 46.70 | C=9.31+46.70 F  *R*^2^=0.97, *p* < 0.01, *n*=63 |

Note: C, physiological capacitance.

**Table S2** The equations and parameters of the model of the leaves Z, R, Xc, Xl with the clamping force (F)

| Parameters-F | Sample | y | k | b | Equation |
| --- | --- | --- | --- | --- | --- |
| Z-F | Control | 0.18 | 0.47 | 0.79 | Z=0.18+0.47 e^(-0.79 F)  *R*^2^=0.99, *p* < 0.01, *n*=63 |
|  | BPH_3h | 0.28 | 0.89 | 0.82 | Z=0.28+0.89 e^(-0.82 F)  *R*^2^=0.99, *p* < 0.01, *n*=63 |
|  | BPH_8h | 0.31 | 1.09 | 0.76 | Z=0.31+1.09 e^(-0.76 F)  *R*^2^=0.99, *p* < 0.01, *n*=63 |
|  | BPH_24h | 0.34 | 1.11 | 0.94 | Z=0.34+1.11 e^(-0.94 F)  *R*^2^=0.99, *p* < 0.01, *n*=63 |
|  | Control | 0.18 | 0.47 | 0.79 | Z=0.18+0.47 e^(-0.79 F)  *R*^2^=0.99, *p* < 0.01, *n*=63 |
|  | SSB_3h | 0.17 | 0.42 | 0.77 | Z=0.17+0.42 e^(-0.77 F)  *R*^2^=0.99, *p* < 0.01, *n*=63 |
|  | SSB_8h | 0.43 | 1.25 | 0.65 | Z=0.43+1.25 e^(-0.65 F)  *R*^2^=0.99, *p* < 0.01, *n*=63 |
| R-F | Control | 0.25 | 0.42 | 0.82 | R=0.25+0.42 e^(-0.82 F)  *R*^2^=0.99, *p* < 0.01, *n*=63 |
|  | BPH_3h | 0.45 | 0.77 | 0.82 | R=0.45+0.77 e^(-0.82 F)  *R*^2^=0.99, *p* < 0.01, *n*=63 |
|  | BPH_8h | 0.56 | 0.77 | 0.78 | R=0.56+0.77 e^(-0.78 F)  *R*^2^=0.99, *p* < 0.01, *n*=63 |
|  | BPH_24h | 0.63 | 0.89 | 0.92 | R=0.63+0.89 e^(-0.92 F)  *R*^2^=0.99, *p* < 0.01, *n*=63 |
|  | Control | 0.25 | 0.42 | 0.82 | R=0.25+0.42 e^(-0.82 F)  *R*^2^=0.99, *p* < 0.01, *n*=63 |
|  | SSB_3h | 0.24 | 0.35 | 0.75 | R=0.24+0.35 e^(-0.75 F)  *R*^2^=0.99, *p* < 0.01, *n*=63 |
|  | SSB_8h | 0.57 | 1.12 | 0.68 | R=0.57+1.12 e^(-0.68 F)  *R*^2^=0.99, *p* < 0.01, *n*=63 |
| Xc-F | Control | 1.12 | 1.97 | 0.68 | Xc=1.12+1.97 e^(-0.68 F  *R*^2^=0.99, *p* < 0.01, *n*=63) |
|  | BPH_3h | 1.15 | 3.23 | 0.74 | Xc=1.15+3.23 e^(-0.74 F  *R*^2^=0.99, *p* < 0.01, *n*=63) |
|  | BPH_8h | 1.25 | 3.35 | 0.68 | Xc=1.25+3.35 e^(-0.68 F  *R*^2^=0.99, *p* < 0.01, *n*=63) |
|  | BPH_24h | 1.27 | 3.46 | 0.81 | Xc=1.27+3.46 e^(-0.81 F  *R*^2^=0.99, *p* < 0.01, *n*=63) |
|  | Control | 1.12 | 1.97 | 0.68 | Xc=1.12+1.97 e^(-0.68 F  *R*^2^=0.99, *p* < 0.01, *n*=63) |
|  | SSB_3h | 1.12 | 1.94 | 0.78 | Xc=1.12+1.94 e^(-0.78 F  *R*^2^=0.99, *p* < 0.01, *n*=63) |
|  | SSB_8h | 1.75 | 4.28 | 0.89 | Xc=1.75+4.28 e^(-0.89 F  *R*^2^=0.99, *p* < 0.01, *n*=63) |
| Xl-F | Control | 1.35 | 1.89 | 0.63 | Xl=1.35+1.89 e^(-0.63 F  *R*^2^=0.99, *p* < 0.01, *n*=63) |
|  | BPH_3h | 1.62 | 3.78 | 0.74 | Xl=1.62+3.78 e^(-0.74 F  *R*^2^=0.99, *p* < 0.01, *n*=63) |
|  | BPH_8h | 1.73 | 3.67 | 0.54 | Xl=1.73+3.67 e^(-0.54 F  *R*^2^=0.99, *p* < 0.01, *n*=63) |
|  | BPH_24h | 1.85 | 4.59 | 0.72 | Xl=1.85+4.59 e^(-0.72 F  *R*^2^=0.99, *p* < 0.01, *n*=63) |
|  | Control | 1.35 | 1.89 | 0.63 | Xl=1.35+1.89 e^(-0.63 F  *R*^2^=0.99, *p* < 0.01, *n*=63) |
|  | SSB_3h | 1.35 | 1.83 | 0.71 | Xl=1.35+1.83 e^(-0.71 F  *R*^2^=0.99, *p* < 0.01, *n*=63) |
|  | SSB_8h | 2.15 | 4.28 | 0.74 | Xl=2.15+4.28 e^(-0.74 F  *R*^2^=0.99, *p* < 0.01, *n*=63) |

Note: R, physiological resistance; Z, physiological impedance; Xc, physiological capacitive reactance; Xl, physiological inductive reactance.

**Table S3** Impact of BPH and SSB infestation on rice electrophysiological parameters

| Sample | IC (pF) | IR (MΩ) | IZ (MΩ) | IXc (MΩ) | IXl (MΩ) |
| --- | --- | --- | --- | --- | --- |
| Control | 21.37±9.17 a | 0.67±0.14 b | 0.65±0.14 b | 3.09±1.65 a | 3.24±1.77 a |
| BPH_3h | 12.61±2.65 a | 1.22±0.36 a | 1.17±0.37 a | 4.38±0.84 a | 5.40±1.05 a |
| BPH_8h | 13.38±4.92 a | 1.33±0.35 a | 1.40±0.16 a | 4.60±1.79 a | 5.40±2.40 a |
| BPH_24h | 11.84±2.50 a | 1.52±0.35 a | 1.45±0.24 a | 4.73±1.19 a | 6.44±2.97 a |
| Control | 21.37±9.17 a | 0.67±0.14 b | 0.65±0.14 b | 3.09±1.65 b | 3.24±1.77 b |
| SSB_3h | 18.03±3.32 a | 0.59±0.05 b | 0.59±0.05 b | 3.06±0.63 b | 3.18±0.61 ab |
| SSB_8h | 9.31±2.31 a | 1.69±0.46 a | 1.68±0.46 a | 6.03±1.35 a | 6.43±1.85 a |

Note: Mean ± standard errors. Different letters indicate significant differences among treatments (*p* < 0.05, one-way ANOVA followed by Duncan’s multiple range test). IC, intrinsic physiological capacitance; IR, intrinsic physiological resistance; IZ, intrinsic physiological impedance; IXc, intrinsic physiological capacitive reactance; IXl, intrinsic physiological inductive reactance.

**Table S4** Impact of BPH and SSB infestation on rice intracellular water metabolism indicators

| Sample | d | IWHC | IWUE | IWHT | IWTR |
| --- | --- | --- | --- | --- | --- |
| Control | 65.87±28.32 a | 105.54±65.93 a | 1.16±1.74 a | 13.32±5.67 a | 7.35±2.23 a |
| BPH_3h | 35.02±5.05 a | 45.51±14.49 a | 0.84±0.28 a | 13.86±1.93 a | 3.47±1.51 b |
| BPH_8h | 43.90±1.75 a | 51.46±27.13 a | 1.18±0.66 a | 18.19±5.31 a | 2.65±0.79 b |
| BPH_24h | 87.58±36.50 a | 41.42±12.46 a | 2.19±0.78 a | 17.70±6.24 a | 2.39±0.16 b |
| Control | 65.87±28.32 a | 105.54±65.93 a | 1.16±1.74 a | 13.32±5.67 a | 7.35±2.23 a |
| SSB_3h | 52.88±14.26 a | 77.58±20.60 a | 0.71±0.17 a | 10.48±1.75 a | 7.31±1.18 a |
| SSB_8h | 52.58±9.36 a | 29.07±10.86 a | 2.13±0.87 a | 14.58±1.34 a | 2.06±0.95 b |

Note: Mean ± standard errors. Different letters indicate significant differences among treatments (*p* < 0.05, one-way ANOVA followed by Duncan’s multiple range test). d, specific effective thickness; IWHC, intracellular water holding capacity; IWUE, intracellular water use efficiency; IWHT, intracellular water holding time; IWTR, intracellular water transfer rate.

**Table S5** Impact of BPH and SSB infestations on rice intracellular nutrient metabolism indicators

| Sample | UNF | UAF | NAC | NTC | NUE |
| --- | --- | --- | --- | --- | --- |
| Control | 0.49±0.22 a | 0.23±0.09 a | 1.82±1.10 a | 3.91±2.52 a | 2.40±0.91 a |
| BPH_3h | 0.50±0.09 a | 0.23±0.06 a | 0.72±0.21 b | 1.62±0.49 b | 2.06±0.32 a |
| BPH_8h | 0.60±0.17 a | 0.28±0.08 a | 0.79±0.41 b | 1.64±0.75 b | 1.86±0.65 a |
| BPH_24h | 0.64±0.24 a | 0.28±0.11 a | 0.67±0.25 b | 1.50±0.51 b | 1.95±1.04 a |
| Control | 0.49±0.22 a | 0.23±0.09 a | 1.82±1.10 a | 3.91±2.52 a | 2.40±0.91 a |
| SSB_3h | 0.37±0.04 a | 0.17±0.01 a | 1.12±0.21 a | 2.40±0.50 a | 2.65±0.44 a |
| SSB_8h | 0.54±0.06 a | 0.27±0.05 a | 0.52±0.17 b | 1.07±0.38 b | 1.87±0.22 a |

Note: Mean ± standard errors. Different letters indicate significant differences among treatments (*p* < 0.05, one-way ANOVA followed by Duncan’s multiple range test). UNF, unit nutrient flux; UAF, unit active flux; NAC, nutrient active transport capacity; NTC, nutrient transport capacity; NUE, nutrient use efficiency.

**Table S6** Impact of BPH and SSB infestations on rice leaf metabolic activity indicators

| Sample | MF | MR | MA |
| --- | --- | --- | --- |
| Control | 44606.00±28827.04 a | 15.34±11.33 a | 8.64±2.69 a |
| BPH_3h | 5602.49±5416.55 ab | 2.80±2.01 b | 4.60±1.35 b |
| BPH_8h | 5585.96±6113.10 ab | 2.41±1.84 b | 4.34±1.52 b |
| BPH_24h | 1935.42±857.09 b | 1.59±0.59 b | 3.73±0.54 b |
| Control | 44606.00±28827.04 a | 15.34±11.33 a | 8.64±2.69 a |
| SSB_3h | 34752.52±13406.45 a | 10.61±3.90 a | 8.30±1.20 a |
| SSB_8h | 1800.48±1712.13 b | 1.22±0.91 b | 3.30±1.02 b |

Note: Mean ± standard errors. Different letters indicate significant differences among treatments (*p* < 0.05, one-way ANOVA followed by Duncan’s multiple range test). MF, metabolic flux; MR, metabolic rate; MA, metabolic activity.

**Table S7-1** Impact of BPH and SSB infestations on rice intracellular dielectric substance transport indicators

| Sample | Kn_R_ | Kn_Z_ | Kn_XC_ | Kn_XL_ | ICR_R_ | ICF_R_ |
| --- | --- | --- | --- | --- | --- | --- |
| Control | 3.24±0.94 a | 3.15±1.01 a | 2.69±0.46 a | 2.63±0.53 a | -0.45±0.19 a | 1.56±0.53 a |
| BPH_3h | 2.39±0.36 a | 2.39±0.38 a | 2.33±0.19 a | 2.33±0.11 a | -1.05±0.38 b | 1.10±0.24 a |
| BPH_8h | 2.94±0.13 a | 2.86±0.20 a | 2.73±0.27 a | 2.40±1.03 a | -1.23±0.45 bc | 1.05±0.17 a |
| BPH_24h | 3.41±0.59 a | 3.47±0.60 a | 3.14±0.41 a | 3.08±0.51 a | -1.71±0.33 c | 1.18±0.29 a |
| Control | 3.24±0.94 a | 3.15±1.01 a | 2.69±0.46 a | 2.63±0.53 a | -0.45±0.19 a | 1.56±0.53 a |
| SSB_3h | 2.39±0.21 a | 2.44±0.23 a | 2.44±0.19 a | 2.20±0.14 a | -0.45±0.06 a | 1.71±0.45 a |
| SSB_8h | 2.23±0.99 a | 2.21±1.03 a | 2.99±0.31 a | 2.35±0.53 a | -2.20±1.43 b | 1.30±0.80 a |

Note: Mean ± standard errors. Different letters indicate significant differences among treatments (*p* < 0.05, one-way ANOVA followed by Duncan’s multiple range test). Kn_R_, transfer number of dielectric substance in response to physiological resistance; Kn_Z_, transfer number of dielectric substance in response to physiological impedance; Kn_XC_, transfer number of dielectric substance in response to physiological capacitive reactance; Kn_XL_, transfer number of dielectric substance in response to physiological inductive reactance; ICR_R_, intracellular conductive resistance of resistive substance; ICF_R_, intracellular conductive force of resistive substance.

**Table S7-2** Impact of BPH and SSB infestations on rice intracellular dielectric substance transport indicators

| Sample | ICR_Z_ | ICF_Z_ | ICR_C_ | ICF_C_ | ICR_L_ | ICF_L_ |
| --- | --- | --- | --- | --- | --- | --- |
| Control | -0.46±0.19 a | 1.65±0.51 a | -3.14±3.45 a | 0.47±0.14 a | -3.33±3.50 a | 0.39±0.10 a |
| BPH_3h | -1.01±0.38 b | 1.16±0.27 a | -3.99±0.91 a | 0.32±0.05 a | -5.22±2.10 a | 0.26±0.03 ab |
| BPH_8h | -1.45±0.22 bc | 1.13±0.18 a | -4.36±2.70 a | 0.30±0.04 a | -4.36±2.95 a | 0.20±0.02 b |
| BPH_24h | -1.61±0.22 c | 1.21±0.26 a | -5.49±2.38 a | 0.36±0.05 a | -9.15±7.30 a | 0.31±0.05 ab |
| Control | -0.46±0.19 a | 1.65±0.51 a | -3.14±3.45 a | 0.47±0.14 a | -3.33±3.50 a | 0.39±0.10 a |
| SSB_3h | -0.45±0.05 a | 1.78±0.45 a | -2.82±0.35 a | 0.40±0.07 a | -3.12±0.18 a | 0.34±0.06 a |
| SSB_8h | -2.15±1.20 b | 1.37±0.82 a | -7.92±3.08 a | 0.35±0.14 a | -8.61±5.52 a | 0.29±0.11 a |

Note: Mean ± standard errors. Different letters indicate significant differences among treatments (*p* < 0.05, one-way ANOVA followed by Duncan’s multiple range test). ICR_Z_, intracellular conductive resistance of total dielectric substance; ICF_Z_, intracellular conductive force of total dielectric substance; ICR_C_, intracellular conductive resistance of capacitive substance; ICF_C_, intracellular conductive force of capacitive substance; ICR_L_, intracellular conductive resistance of inductive substance; ICF_L_, intracellular conductive force of inductive substance.

**Table S8-1** Impact of BPH and SSB infestations on energy metabolism indicators of various intracellular dielectric substances in rice

| Sample | ΔG_R-U_ | ΔG_R_ | ΔG_Z-U_ | ΔG_Z_ |
| --- | --- | --- | --- | --- |
| Control | 5.16±2.64 a | 346.82±226.10 a | 4.70±2.18 a | 311.15±181.20 a |
| BPH_3h | 2.65±0.56 a | 95.56±31.10 b | 2.63±0.51 a | 94.46±29.25 b |
| BPH_8h | 3.20±0.53 a | 140.03±19.89 b | 2.61±0.18 a | 114.84±12.52 b |
| BPH_24h | 2.94±0.69 a | 275.73±148.96 a | 3.05±0.86 a | 285.35±154.50 a |
| Control | 5.16±2.64 a | 346.82±226.10 a | 4.70±2.18 a | 311.15±181.20 a |
| SSB_3h | 3.13±0.31 a | 156.09±47.56 b | 3.13±0.37 a | 156.61±49.14 a |
| SSB_8h | 2.06±0.72 b | 114.39±58.25 b | 1.70±0.19 b | 91.12±25.80 b |

Note: Mean ± standard errors. Different letters indicate significant differences among treatments (*p* < 0.05, one-way ANOVA followed by Duncan’s multiple range test). ΔG_R-U_, unit metabolic energy based on resistance; ΔG_R_, metabolic energy based on resistance; ΔG_Z-U_, unit metabolic energy based on impedance; ΔG_Z_, metabolic energy based on impedance.

**Table S8-2** Impact of BPH and SSB infestations on energy metabolism indicators of various intracellular dielectric substances in rice

| Sample | ΔG_Xc-U_ | ΔG_Xc_ | ΔG_Xl-U_ | ΔG_Xl_ |
| --- | --- | --- | --- | --- |
| Control | 3.17±0.99 a | 207.55±100.46 a | 3.41±1.46 a | 230.70±144.88 a |
| BPH_3h | 2.37±0.28 a | 84.16±20.63 b | 2.36±0.39 a | 84.46±24.13 b |
| BPH_8h | 3.06±0.38 a | 134.22±16.66 b | 3.24±0.32 a | 142.78±19.16 b |
| BPH_24h | 2.83±0.86 a | 266.02±150.13 a | 2.47±0.62 a | 236.56±147.85 a |
| Control | 3.17±0.99 a | 207.55±100.46 a | 3.41±1.46 a | 230.70±144.88 a |
| SSB_3h | 2.62±0.18 a | 133.50±48.61 b | 2.37±0.13 a | 119.63±40.34 a |
| SSB_8h | 2.43±1.02 a | 135.97±78.06 b | 2.13±0.76 b | 117.57±60.60 a |

Note: Mean ± standard errors. Different letters indicate significant differences among treatments (*p* < 0.05, one-way ANOVA followed by Duncan’s multiple range test). ΔG_Xc-U_, unit metabolic energy based on capacitive reactance; ΔG_Xc_, metabolic energy based on capacitive reactance; ΔG_Xl-U_, unit metabolic energy based on inductive reactance; ΔG_Xl_, metabolic energy based on inductive reactance.

**Table S9** Parameters in transgenic rice overexpressing planthopper-derived elicitors

| Category | Indicator | Control | Myosin_OE_4 | PDI1_OE_9 |
| --- | --- | --- | --- | --- |
| Electrophysiological parameters | IC | 68.70±19.08 | 17.42±4.26 ** | 14.7±3.08 ** |
|  | IR | 0.40±0.27 | 7.96±2.54 ** | 4.19±2.4 ** |
|  | IZ | 0.36±0.22 | 3.41±1.13 ** | 2.94±1.06 ** |
|  | IXc | 0.72±0.29 | 3.58±1.04 ** | 4.34±1.01 ** |
|  | IXl | 1.23±0.68 | 8.89±1.53 ** | 7.21±1.85 ** |
| Intracellular water metabolism indicators | d | 162.83±36.98 | 72.34±23.21 ** | 36.03±13.99 ** |
|  | IWHC | 606.96±179.71 | 77.35±27.75 ** | 60.97±25.28 ** |
|  | IWUE | 0.37±0.24 | 1.21±0.50 * | 0.77±0.36 |
|  | IWHT | 18.97±3.78 | 45.04±15.48 * | 34.67±8.8 * |
|  | IWTR | 36.77±27.83 | 2.37±2.11 * | 1.89±1.57 * |
| Intracellular nutrient metabolism indicators | UNF | 0.69±0.14 | 2.58±1.49 * | 1.46±0.24 * |
|  | UAF | 0.30±0.04 | 0.73±0.28 * | 0.56±0.34 |
|  | NAC | 10.03±6.41 | 1.24±0.81 * | 1.00±0.74 * |
|  | NTC | 22.18±13.72 | 3.50±1.43 * | 2.48±1.72 * |
|  | NUE | 1.49±0.29 | 0.56±0.32 ** | 0.7±0.11 ** |
| Leaf metabolic activity indicators | MF | 6345859.46±73265.31 | 6726.75±1431.68 ** | 3151.48±504.6 ** |
|  | MR | 546.95±168.36 | 4.64±1.15 ** | 3.05±4.39 ** |
|  | MA | 30.14±10.49 | 3.83±1.89 ** | 3.37±1.16 ** |
| Intracellular dielectric substance transport indicators | Kn_R_ | 2.96±0.45 | 4.07±0.57 | 3.03±0.7 |
|  | Kn_Z_ | 2.91±0.41 | 3.97±0.65 * | 1.62±1.18 |
|  | Kn_XC_ | 2.74±0.28 | 3.25±0.26 * | 2.64±1.41 |
|  | Kn_XL_ | 3.00±0.59 | 3.26±0.40 | 3.52±1.43 |
|  | ICR_R_ | -0.29±0.27 | -12.66±1.43 ** | -1.94±0.61 * |
|  | ICF_R_ | 3.00±1.13 | 0.43±0.29 ** | 0.17±0.09 ** |
|  | ICR_Z_ | -0.36±0.31 | -2.52±2.46 | -1.34±0.99 |
|  | ICF_Z_ | 3.25±1.16 | 0.52±0.25 ** | 0.23±0.1 ** |
|  | ICR_C_ | -0.56±0.32 | -2.41±1.19 * | -1.94±1.27 |
|  | ICF_C_ | 1.07±0.35 | 0.29±0.07 * | 0.15±0.05 |
|  | ICR_L_ | -0.64±0.39 | -10.29±3.44 ** | -3.15±0.62 ** |
|  | ICF_L_ | 0.82±0.32 | 0.21±0.09 * | 0.09±0.04 ** |
| Energy metabolism indicators of various intracellular dielectric substances | ΔG_R-U_ | 4.78±1.14 | 4.09±1.97 | 6.22±2.3 |
|  | ΔG_R_ | 809.63±371.65 | 298.95±152.44 * | 284.56±155.74 * |
|  | ΔG_Z-U_ | 4.65±1.04 | 5.11±2.67 | 5.36±2.12 |
|  | ΔG_Z_ | 786.77±350.96 | 368.72±186.59 | 263.25±177.47 |
|  | ΔG_Xc-U_ | 4.53±0.69 | 5.11±1.11 | 7.45±3.19 |
|  | ΔG_Xc_ | 738.36±204.88 | 337.63±193.55 | 404.67±323.77 |
|  | ΔG_Xl-U_ | 5.31±1.10 | 3.55±0.86 * | 5.56±2.9 |
|  | ΔG_Xl_ | 856.65±205.37 | 262.02±110.79 ** | 212.48±113.18 ** |

Note: Mean ± standard errors. Asterisks indicate significant differences among treatments (* *p* < 0.05; ** *p* < 0.01).
